# A qualitative study of user perceptions of mobile health apps

**DOI:** 10.1186/s12889-016-3808-0

**Published:** 2016-11-14

**Authors:** Wei Peng, Shaheen Kanthawala, Shupei Yuan, Syed Ali Hussain

**Affiliations:** 1 Department of Media and Information, Michigan State University, 404 Wilson Road, Room 409, East Lansing, MI 48824 USA; 2 Department of Advertising & Public Relations, Michigan State University, 404 Wilson Road, Room 309, East Lansing, MI 48824 USA; 3 School of Journalism, Michigan State University, 404 Wilson Road, Room 359, East Lansing, MI 48824 USA

**Keywords:** Mobile apps, Smartphone, Technology acceptance, Qualitative study, Self-regulation, Health promotion, mHealth, Adoption, User perception

## Abstract

**Background:**

Mobile apps for health exist in large numbers today, but oftentimes, consumers do not continue to use them after a brief period of initial usage, are averse toward using them at all, or are unaware that such apps even exist. The purpose of our study was to examine and qualitatively determine the design and content elements of health apps that facilitate or impede usage from the users’ perceptive.

**Methods:**

In 2014, six focus groups and five individual interviews were conducted in the Midwest region of the U.S. with a mixture of 44 smartphone owners of various social economic status. The participants were asked about their general and health specific mobile app usage. They were then shown specific features of exemplar health apps and prompted to discuss their perceptions. The focus groups and interviews were audio recorded, transcribed verbatim, and coded using the software NVivo.

**Results:**

Inductive thematic analysis was adopted to analyze the data and nine themes were identified: 1) barriers to adoption of health apps, 2) barriers to continued use of health apps, 3) motivators, 4) information and personalized guidance, 5) tracking for awareness and progress, 6) credibility, 7) goal setting, 8) reminders, and 9) sharing personal information. The themes were mapped to theories for interpretation of the results.

**Conclusions:**

This qualitative research with a diverse pool of participants extended previous research on challenges and opportunities of health apps. The findings provide researchers, app designers, and health care providers insights on how to develop and evaluate health apps from the users’ perspective.

**Electronic supplementary material:**

The online version of this article (doi:10.1186/s12889-016-3808-0) contains supplementary material, which is available to authorized users.

## Background

Currently over 97,000 health-related apps (termed as health apps hereafter) are available in the health and fitness category of the Apple app store and Google play store, with about 1000 more being created every month. This number is expected to increase by about 25% each year [1]. These health apps include medical apps for health providers and medical education, patient-centered apps for disease management or self-diagnosis, and general health and fitness apps for lifestyle management [2]. With the exponential increase of health apps, research on their use in the areas of health promotion and disease management has also increased significantly over the past 5 years. There have been a number of intervention studies based on mobile apps and most of these focused on specific medical issues such as diabetes [3, 4], pain management [5], weight loss [6, 7], etc. The intervention studies mostly focus on the patient population using mobile apps for treatment or disease self-management [3–7].

However, a majority of the available health apps are for health and wellness promotion and disease prevention for the general public. A large number of the studies on such health apps adopted a content analysis approach [8–14]. Although these content analyses provide important insight into what apps are available and whether theories and evidence-based practice inform app design, qualitative studies examining these health apps from the users’ perspectives are limited. It is important to go beyond the content analysis to examine users’ perceptions of these off-the-shelf health apps to gauge the differences between users who have, who will and who never will adopt mHealth [15]. By examining users’ experience with current apps, researchers and app developers can better design future mhealth interventions to be both effective and accepted by end users.

Among the limited qualitative research on health apps, one focus group study examined user perceptions of mobile apps in supporting behavior change and provided insights from users’ perspective for researchers and designers to better design and develop health apps [16]. Specifically, this study found that healthy young adults were interested in using health apps to support health-related behavior change. Potential users expected health apps to be accurate, legitimate, secure, and able to record and track behavior and goals. They also expected health apps to require minimal effort to operate and have the ability to acquire advice and information “on the go”. These healthy young adults did not like apps with context-sensing capabilities or social media features. Despite insightful findings, one limitation of the study is that its participants are biased toward the younger population (average age was 23.8) with relatively high education and income (affiliated with a university). The most recent data from the Pew Internet Research indicate that 54% of Americans ages 50–64, 52% of Americans with less than college-level education, and 50% of Americans with less than $30,000 annual household income own a smartphone [17]. Smartphone penetration rate is over 80% for the 25 to 54 years old age group in the United Kingdom [18]. The data indicate that smartphones and health apps are not just the products used by young people with high education and high income. Therefore, it is important to extend the previous research by examining a more diverse pool of users with various age groups and social economic status to better understand users’ perception of these apps.

Another limitation of existing qualitative studies on health apps is the lack of a systematic framework to guide the qualitative exploration. Although the qualitative research on user perception is exploratory in nature, having a systematic framework helps the research stay focused. Therefore, the current research attempts to systematically examine users’ views about features and capabilities of health apps, based on the mobile phone features and intervention strategies identified in Klasnja and Pratt’s previous research [19]. Specifically, their framework lays out five behavioral intervention strategies that can be achieved via various phone functions (i.e., text messaging, cameria, native applications, automated sensing, and Internet access), which include: 1) tracking health information, 2) involving the health care team, 3) leveraging social influence, 4) increasing the accessibility of health information, and 5) utilizing entertainment.

Additionally, although this qualitative research takes a bottom-up inductive approach, we discuss the findings in light of theoretical frameworks (e.g., extended unified theory of acceptance and usage of technology (UTAUT2) [20], self-regulation [21]), which pave the road for future quantitative research to systematically adopt theoretical frameworks for a top-down deductive approach. In other words, theoretical frameworks did not guide this qualitative work, as qualitative research usually takes the bottom-up inductive rather than the top-down deductive approach to analyze results. Instead, existing theoretical frameworks were brought in for the interpretation of findings because they can help explain the how and why of the findings as well as abstract the findings to guide future research. In the following section, we explain the procedure for conducting the current qualitative research. Inductive thematic analysis was used to analyze transcripts leading to the identification of nine themes. The discussion provides insights for researchers and designers to better design and implement apps for health promotion and behavior change.

## Methods

### Participants

To make sure we adhered to qualitative reporting standards, we followed the 32-item consolidated criteria for reporting qualitative studies (COREQ) checklist (Additional file 1). A total of 44 individuals, including 39 in six focus groups and five in interviews participated in this study. Purposive sampling was used and recruitment was not based on data saturation. All the consented participants completed the study. Inclusion criteria were the ownership and regular use of a smartphone. To get a well-rounded picture about why people used or did not use health apps, both individuals with and without prior knowledge or usage of health apps were included. Each focus group consisted of three to nine participants. Two focus groups consisted of 17 college students (11 female) were recruited from the subject pool in a large Midwestern university in the U.S. Four other focus groups were formed with 22 non-student participants (18 female) through email to staff members at the same university. Although the focus groups in this study aimed to address the limitation of previous studies by including participants with a wide age range, the educational level of the participants was still relatively high. In an attempt to include a more diverse pool, we also recruited five participants (one female) via solicitation at a local plaza. These five participants were not affiliated with the university and were of diverse social economic statuses. However, due to their scattered working schedules, we were unable to conduct a focus group and individual interviews were conducted instead. The variation in profession type among the interviewees (i.e., automobile mechanic, truck driver, hair stylist, small business owner, morning shift as medical assistant and night shift at fast food franchise) provided information about how individuals with relatively less education and physically strenuous working-hours use health apps. In general, all the participants considered themselves to be in either ‘very good health’ or ‘good health’ condition, had an average health consciousness score of 3.8 out of 5 (assessed by the health consciousness scale [22]), and had never used medical apps (which are more medically focused as compared to the health apps of our study). Table 1 summarizes the demographic information and app usage of the participants. The data analysis combined the six focus groups and five interviews.

**Table 1:** Participant demographics and smartphone and app usage

All participant demographics and smartphone and app usage		
Gender		
Female		*n* = 30 (65%)
Age^a^		*M* = 37.2, SD = 15.7
18–25 years old		*n* = 17 (40%)
26–50 years old		*n* = 15 (36%)
Over 50 years old		*n* = 10 (24%)
Race		
Caucasian		*n* = 27 (61%)
Asian		*n* = 10 (23%)
African American		*n* = 6 (14%)
Hispanic		*n* = 1 (2%)
Participants with prior health app usage^b^		*n* = 25 (57%)
Phone type		
iOS		*n* = 27 (61%)
Android		*n* = 15 (34%)
Blackberry		*n* = 2 (5%)
Average number of apps^c^		31–40
Average length of smartphone usage		32 months
Average number of apps used weekly^d^		6–10
Average daily app usage^e^		61–90 min
Participants who never paid for an app		*n* = 34 (77%)
Characteristics of focus group participants		
	Students	Non-Students
Number of focus groups	2	4
Gender	11 female; 6 male	18 female; 4 male
Age	18–23 years	30–67 years
Education level	Some high school to post graduate education	
Characteristics of interview participants		
Number of interviews	5	
Gender	1 female; 4 male	
Age	34–56 years	
Education level	Some college or technical schooling	

^a^Two participants did not disclose age

^b^Although after later 2014, most smart phones have pre-installed health apps, data of this study was collected in early 2014, and thus almost half of the participants did not have health apps

^c^Participants were asked to indicate number of apps they owned based on a provided scale. This scale included a range of number of apps (for example: 1–5, 6–10, etc.) This number indicates the average range selected by participants

^d^Participants were asked to indicate number of apps used weekly based on a provided scale. This scale included a range of number of apps (for example: 1–5, 6–10, etc.) This number indicates the average range selected by participants

^e^Participants were asked their average daily app use based on a provided scale. This scale included a range of minutes (for example: 0–30, 31–60, etc.) This number indicates the average range selected by participants

### Procedure

The institutional review board at Michigan State University approved the study. After acquiring their consent, each participant first completed a questionnaire about demographics, smartphone and mobile app usage, and health status. The second author (female) served as the moderator and the third author (female) recorded the sessions and took notes. The fourth author (male) conducted the interviews. Both the moderator and the interviewer were doctoral students with a Master’s Degree and were trained with qualitative research methods. The moderator and the interviewer did not have prior relationship with the participants. Nobody else besides the authors and participants were present during data collection. All the authors had a positive attitude towards health apps, but the authors strived to remain neutral in the conversations with participants. Each focus group session took place in a conference room and ran for about 40 to 90 min. Non-student participants were provided with a free meal and a $20 gift card for their time and student participants were provided with a free meal and extra course credit. The interviews were conducted by the last author, at participants’ workplace, home or a nearby café. Each interview lasted for 30–45 min. The interviewees were provided a $40 gift card as incentive. All focus groups and interviews were audio-recorded and then transcribed verbatim. The anonymized transcriptions supporting the conclusions of this article are available at goo.gl/T9oZvk.

The moderator and the interviewer followed a discussion guide developed jointly by the authors (see Additional file 2) to direct the conversation. Participants were first provided an overarching introduction about the purpose of the study. They were then asked questions about their overall app usage, knowledge about health apps and their usage, and reasons for liking or disliking apps, including health apps. Participants freely discussed their own experiences without prompts.

*Trigger materials*. Next, in order to educate participants with no health apps and enlighten others about the wide variety of health apps, participants were exposed to a set of trigger materials (see Additional file 3). These materials included screen captures of various features of health apps based on Klasnja and Pratt’s framework [19] of the five behavioral intervention strategies enabled by smartphones: 1) tracking health information (a: goal setting, b: behavior monitoring and tracking, c: reminders, d: progress visualization), 2) involving health care team (e: sensing and information sharing with health care providers), 3) leveraging social influence (f: social networking), 4) increasing the accessbility of health information (g: information such as tips, coaching, etc), and 5) utilizing entertainnment (h: use of entertainment, i: use of gamification). The participants were exposed to the above categories and sub-categories of health apps, one at a time, and asked to discuss their thoughts about them. Since our study focused on the general usage of health apps in users’ day-to-day lives, we did not include any medical apps in the trigger materials, which would be disease specific or apps connected to medical devices or for provider communication [2, 23]. They were asked to go into as much detail as possible, explain what they liked or disliked, whether or not they had used that particular or a similar app before and what led them to continue or discontinue using it. The participants who did not have such an app were asked to provide reasons for non-use.

### Data analysis

The verbatim transcripts were coded using the software NVivo. Inductive thematic analysis [24] was adopted to analyze the data. The focus group and interview transcriptions were analyzed as a whole. Each recording was coded separately by at least two authors who independently came up with labels to attach to text segments that appeared to indicate important user perspective. Then the team came together to compare their codes and revise the codes in an iterative fashion to develop a set of themes that captured the essence of the focus group discussions or interviews. Finally, the raw data were compared with the emerging theme labels and definitions, and further refined by merging, adding, and removing redundant themes. At the end, nine themes were identified. The results section describes them with illustrative quotations.

## Results

### Barriers to Adoption of Health Apps

Among the participants, only 57% had health apps. Four types of reasons were identified by the participants explaining why they did not *adopt* or *start* to use health apps.

*Low Awareness of health apps*. More than a quarter of the participants were unaware of or did not even think that health apps were available. *Lack of need for health apps*. Participants did not feel the need to use a health app either because other tools were already available (e.g., notebook, spreadsheet, a website which performed a similar function as the app) and it was not necessary to use a mobile app or they already had formed healthy habits and a tool was no longer needed (e.g., a physically active person would not need an app to motivate them to exercise). Although they did not consider a health app a necessity for themselves, they thought that a health app might be helpful for certain individuals such as patients or people who need the apps for motivation or to establish healthy habits.

“I will just go to the website. I don’t know the benefit of this app. I have to see what the benefit will be.” (Male, non-student, focus group 6 [MNS, FG6])

“I heard they more focus on people’s chronic issues. I don’t really see them useful in [my] life.” (MNS, FG4)

“I do regular exercise, like dancing, so I don’t need to track the calories.” (Female college student [FCS], FG2)

*Lack of app literacy*. Participants did not know which app to choose or how to use it. One female college student stated that “health app is only developed [in] recent years. Consumers might not have a clear definition what is [a] good health app. There are so many functions. It is hard for consumers to decide. [They are] not sure which one fits; [they] eventually will just give up”.

*Cost* was identified as an important determining factor for app adoption, across all the participants in all age groups and social economic status. Among the participants, 77% used only free apps. However, this does not mean that people are not willing to pay for apps. Participants indicated that they would pay, usually a small amount of one dollar or less, if they found the app worth buying. In other words, only if the app had highly unique functions and features, not normally found in free apps, then they would consider buying it.

“It depends on what the app is…what it can do…if a free app can do the same thing then I don’t see the point…” (FCS, FG2)

“Free is important but also working smoothly and free.” (Female, non-student [FNS], FG5)

### Barriers to Continued Use of Health Apps

*Lack of time and effort*. Participants who had health apps indicated a number of barriers to their *continued use*. The primary reason was the required time and effort. One male non-student participant said: “You have to individually [manually] input everything. Doing that, that’s a lot of time.” Ease of use and simplicity is thus one of the top desired features among the participants to overcome the barrier.

“Ease of use. You want to get to the process as simple as possible. The app doesn’t have to do *everything*, but you need to feel easy to use” (MNS, FG4)

“Some apps are so complicated. I delete ‘em, because it’s just too much, you know. So I’m looking for something pretty straightforward. It does what you need it to do without 10 million different things to do one thing.” (Male truck driver in interview)

*Lack of motivation and discipline*. The participants felt that unless an individual was already motivated or had the discipline or dedication, it would be hard for them to continue using health apps.

“It just takes a lot of discipline.” (FNS, FG6)

“Someone who is using this one [a dietary tracking app] is kind of dedicated.” (Male college student [MCS], FG4)

Additional barriers to adoption and continued use of health apps brought up by the participants included radiation from the cell phone, apps that take storage space of the phone or drain battery, and the phone being carried in the purse and thus not used enough.

### Motivators to Use Health Apps

Motivators are internal and external factors that help health app users either start or continue to use them.

*Social competition*. One of the external motivators was seeing other people using the app and sharing behavioral data that could be compared to others on social networking sites. Participants considered this comparison or ‘competition’ as a double-edged sword: it could be helpful and motivating for certain individuals in certain contexts yet it may be demotivating and backfire in other situations, especially when individuals fear that they are too far behind compared to their friends or family.

“Seeing a family member run and I know I have to do it, so it keeps you motivated to do it.” (FCS, FG2)

“I think it would be a mix, depending on the stage. If you see someone [running], they may encourage you or discourage you. I think it just depends on where your personality is....... I think it is good on one hand, but on the flip side, it could discourage people who tried to get started on that.” (FNS, FG3)

*Intangible Rewards*. Other apps provide users with virtual badges they could obtain or levels they could unlock. These intangible rewards are intended to motivate people by triggering their competitive natures.

“Earning badges [was] important when I was doing it…We learned as a kid, to consider [it] as [an] accomplishment.” (FNS, FG3)

*Tangible rewards*. While badges, levels and motivational messages from an app provide intangible rewards, some individuals prefer a tangible one.

“Money is one of the biggest motivators.” (FNS, FG3)

“I like the rewards [i.e., badges] on there [inside the app], but it’s nothing I can touch and feel like a real reward. So for me, if this app was saying once you reach 200 miles or 150 miles, you get a free t-shirt…things like that, [it will be motivating]. Rewards are good but only if you can feel and touch them.” (Male truck driver, interview)

*Hedonic factor*. Adding a gaming element or entertainment to an app was motivating for a certain group of people. However, most of the participants did not consider the entertainment element critical for adults using health apps. On the other hand, they assumed that a health app with fun elements might be a positive thing for children as long as they did not see through the game aspect of an app and know it was something being taught to them.

*Internal dedication and motivation*. Intrinsic factors are not motivations provided by an app, but factors internally prompting a person to use the app. Some participants indicated that an app could only do so much. Ultimately, it is an individual’s internal dedication and motivation that will determine whether they would continue to use it for health enhancement or behavior change.

“Maybe, they need to be given that [app], but eventually, it is an internal [thing]. People are motivated from inside out.” (FNS, FG3)

“These apps will help you only so far. Then you have to take physical initiative on yourself.” (Male shop owner, interview)

### Information and Personalized Guidance

Most participants who liked having health apps stated that what they liked most was its ability to provide information, which they would not have otherwise. Some of the information might be directly provided by the app, some of the information might be user generated and shared on the app. They preferred to have diverse information so even individuals with special needs may be able to find relevant information.

“WebMD [app] gives a lot of information” (FNS, FG6)

“I like that it gives you routes that other people use [to run], so you can use them too” (FCS, FG2)

“Does it have different category, gluten free?” (FNS, FG3)

“How ethnically diverse are these [food] options?” (FNS, FG5)

In particular, they valued personalized and tailored information. One male college student said: “It could be an app that you type in personal info, your weight, blood pressure, then comes out a personal plan.” Similarly, the male truck driver said: “I like the idea of this app making things simple for me, telling me what to eat, my goal weight…It’s more tailored for you and it makes things easy.”

While many people have the motivation to carry out healthy activities, sometimes they may not know the right way to go about it. Some of the information from the health apps guided people to places with more healthy food choices, or provided instructions of how to be healthy. Therefore, many participants liked apps with personalized coaching and guidance with specific plans.

“I need the instructions, [for instance], you need to lift 80 lb, 5 times to reach this goal. So for me tracking what I do is good, but I still am looking for something that’s telling me what I need to do to get to that end goal, knowing whether it’s gain mass, or lose fat. I like to know that I’m going there and doing the right thing to achieve this goal.” (Male truck driver, interview)

“I went to a conference in New Orleans. I need vegetable that is not covered in gravy, or fried. Where can I go? So it [having an app with healthy options] is useful.” (FNS, FG3)

On the other hand, participants did not like the idea of having to take orders or suggestions from a machine. In certain situations, for example, when they lack the ability to carry out a particular activity till the end or fail to reach their set goal, having the app continue to give negative feedback may demotivate and backfire.

“I don’t want an electronic device telling me what to do.” (FNS, FG5)

“The progress I didn’t make—it shows [and thus is demotivating].” (Female medical assistant/fast food store worker, interview)

### Tracking for Awareness and Progress

Many health apps have a built-in feature to track the user’s activity, including diet, exercise, etc. For example, users input their food intake and the app creates a log of what has been consumed on a particular day and provides the user with feedback, such as, their estimated weight loss or gain if they continue to eat in the same pattern over the next few days or how far they are from their goal weight. Most participants liked having the tracking feature in their apps because this type of self-monitoring increases their awareness. Additionally, the tracked data could be reviewed retrospectively so they were able to observe their progress, especially, when the progress was also visualized in a graphic manner for easy interpretation.

“It helps you to keep track of your calories and helps keep you under budget of what calories you want” (Female medical assistant/fast food store worker, interview)

“You get a chance to see what you do on a daily basis, something you’re probably not aware of.” (Male truck driver, interview)

“If I could see my progress over the day, it’s like easier to see than just look back and say ‘what did I do yesterday?’” (FCS, FG2)

However, the tracking feature did backfire in some cases. People might not be faithful to themselves and the app and cheat the tracking app.

“A lot of times people just try to lie to the app about the amount they ate. You just don’t put everything you ate. It is not helping anybody.” (FCS, FG4)

Even though tracking was a very positively favored characteristic of health apps, some participants thought its usefulness was temporary. After tracking for a while to learn about their own routines, they no longer needed to track themselves anymore. Therefore, tracking was perceived to be helpful for users to gauge everyday behavior pattern initially or for people who was undergoing changing their current behaviors for close monitoring. However, since the purpose of such a health app would be to educate oneself about the tracked health behavior, such an action can be looked upon as a positive thing. The user in this scenario would have successfully learned about themselves and therefore do not need to use the app anymore.

“After I learned the initial…what on average I’m putting in every day, I would lose interest in it.” (Male hair stylist, interview)

“I think for the most part your days would be relatively the same” (FNS, FG6)

Many participants liked sensor-based automatic tracking, such as the accelerometer, which tracks a person’s footsteps each day. The automatic tracking may partially resolve the issue of unreliable self-reported data in tracking. One male non-student participant indicated that having an app to automatically sense how much he ate or even his mood would be good. However, at the time of this study, there was no standard in the market to evaluate how accurately the sensing or tracking systems worked and participants had concerns about reliability of the automatically tracked information.

“[I like that] you don’t have to count your steps; it will do it for you” (MCS, FG2)

“Personally, I have a bias against it [sensor] because I don’t think it would be very accurate, so I don’t use it.” (FCS, FG2)

“It’s really not that accurate because, I just run on a treadmill so I always get different results from what the treadmill says [and what the app says].” (MCS, FG2)

### Credibility

To solve the accuracy concerns we discussed above regarding reliability of automatic sensors, participants mentioned several ways to evaluate the credibility of a health app before downloading it. Users normally perceived friends’ recommendations as highly trustworthy.

“Most of mine [my apps] are friend recommendations, people with similar activities.” (FNS, FG5)

Some participants reported that they would make the decisions based on the comments or reviews from others (non-friends), such as the ranking of the app in the market or the rating by other users.

“I will look for reliable ratings so I know which one would be most popular…I tend to go to that one. (FCS, FG6)

Participants also relied on sources which they already trusted or were familiar with to judge app credibility. A female college student said: “Sometimes the app is branded……so I trust it.” Similarly, a male non-student participant said: “More likely to use an app by organizations that I am familiar with, not [a commercial] company.”

Some participants would not rely on a single source to evaluate app credibility. They might cross validate the app by checking with other sources, such as their doctors. Besides cross checking with other sources, another way to test credibility, which was also mentioned by the majority of the participants, was testing the app by trying it out. Because the majority of health apps are free or offer trial versions, users have the opportunity to try the app and see whether it lives up to their expectations.

“Like the *Babybump* one [an app the participant has used]…I’ll talk to my doctor or whatever and I’ll read things… It’s the same as *MapMyFitness*. You kind of know if it’s lying because over time you can compare that clearly I’m not burning as many calories on the treadmill as I am in real life…” (FNS, FG6)

### Goal Setting

In addition to the information and tracking features in health apps, many participants also liked the goal-setting feature in many apps. They believed that goal setting, especially small daily and weekly goals, could help them discipline themselves and slowly change their behaviors. Some participants also indicated that goal setting would work well with tracking, real time feedback and progress report.

“I’m not good at self-discipline and exercise, so maybe this [goal setting in the app] can help me get to my goal.” (FCS, FG6)

“Maybe in the beginning of the week, like ‘you can do it’, middle of the week, ‘how are you doing’, end of the week, ‘how did you do’.” (FNS, FG3)

### Reminders

Another common feature, liked by most participants, was the reminder. Reminders were found particularly useful for busy individuals who tended to forget things or who had the need to remember multiple medications in a given day. Although reminders are available in other tools as well, participants found mobile apps to be one of the most convenient. However, some participants also pointed out that the timing and frequency of the reminders or push notifications needed to be well designed to be customizable, because otherwise users would just ignore them.

### Sharing Personal Information

Many mobile apps have social networking features and enable sharing personal information, such as physical activity patterns, miles run and routes, food intake, or weight. By sharing information with family or friends, individuals might receive informational and emotional social support. At the same time, shared information from family or friends, might also fuse social competition. Many participants, including both the young and the old, female and male, high and low social economic status individuals, were reluctant to share personal information. The primary reason for not wanting to share personal information was because people considered health-related information such as exercise and dietary patterns as private matters, which did not warrant sharing with others. The participants not only disliked sharing their own, they even expressed annoyance when seeing their friends share such information on social networking sites.

“I don’t really care if people know what I did at the gym. I’m sure they don’t either, so it’s kind of a personal thing.” (MCS, FG2)

“I don’t want [my exercise data] on Facebook, only want to know about my own progress.” (FNS, FG3)

Another reason for not wanting to share personal information was the concern of how the information might be exploited by a third party, such as health insurance companies or advertisers. This concern was most prominent among non-student participants. Some were even concerned that the information (e.g., routine running routes) could put individuals in danger.

Although most of the participants were not favorable to sharing personal information, they indicated that they would share some information, depending on necessity and security as well as whether they could control with whom they would share and what type of information was shared. They preferred to share the information with a small group of individuals designated by themselves. Some of them would not share information with strangers yet some were open to the idea.

“Calorie intake. Starting weight. Ending weight. That would be the things I would share only if I chose to with certain people.” (Male hair stylist, interview)

“The only concern is who has my medication info. If it is secure, I will think it is helpful.” (MNS, FG4)

“I am more likely to interface with strangers. I have done some fitness goal with this community of strangers that are doing the same thing, more comfortable with that.” (FNS, FG3)

“You need real personal contact to do it [providing social support based on the shared information]. I don’t find they [strangers online] are accountable the same way like one-on-one contact.” (MNS, FG4)

## Discussion

A qualitative study about health app user perceptions was conducted with a diverse pool of participants of various age groups and social economic status. Our findings add evidence to the understudied area of user experience and perspectives on health apps. Via six focus groups of 39 participants and five individual interviews, we identified barriers faced by smartphone users for adoption and continued use of health apps. Through their own experiences and by showing participants various common features of health apps, we summarized the motivating or facilitating features that drive health app usage, as well as factors hindering their use (Table 2). Many of the themes we identified were consistent with Dennison et al.’s findings [16], including smartphones as valuable information sources, tracking progress and raising awareness, aversion of sharing information, privacy and security concerns, skepticism over context sensing, necessity for efficiency and convenience, credibility and accuracy. The challenges to use and continue to use health apps faced by our diverse pool of participants were consistent with Dennison et al. [16]. The primary barrier to continuing using these apps was the required time and effort.

**Table 2:** Summary of identified themes and sub-themes mapped to theoretical constructs

Theme/Subthemes	Construct in theories
Factors hindering health app use	
Low awareness of health apps	N/A
Lack of need for health apps	Performance expectancy^a^; perceived usefulness^b^; perceived behavioral control^c^; compatibility^d^; outcome expectations^e^
Lack of app literacy	Facilitating conditions^a^; perceived ease of use^b^; ease of use^c^
Cost	Price value^a^
Lack of time (and effort)	Effort expectancy^a^
Lack of motivation and discipline	
Factors driving health app use	
Social competition	Social influence^a^ descriptive norms^b,c,d^ subjective norms^b,c,d^; visibility^e^
Intangible rewards	Self-reactance^f^
Tangible rewards	Self-reactance^f^
Hedonic factor	Hedonic motivation^a^
Internal dedication and motivation	N/A
Information and personalized guidance	Modeling^f^; Tailoring^g^
Tracking for awareness and progress	Self-observation^f^. self-regulation^h^
Credibility	N/A
Goal setting	Goal setting theory^i^
Reminder	Cues to action^j^
Sharing personalized information	N/A

^a^Extended Unified Theory of Acceptance and Use of Technology [20]

^b^Technology Acceptance Model [31]

^c^Theory of Reasoned Action [32]

^d^Theory of Planned Behavior [33]

^e^Innovation Diffusion Theory [34]

^f^Social Cognitive Theory [21]

^g^Tailoring [28, 29]

^h^Self-Regulation Theory [35]

^i^Goal Setting Theory [36]

^j^Health Belief Model [37]

We also discovered many new findings among our participants who were older and of lower social economic status. For instance, low app awareness and low app literacy are two factors leading to non-adoption, suggesting that increasing awareness and educating the general public about health apps is needed in order to make mHealth widely accepted. Users liked information from the apps, but more importantly, they desired personalized information. Although many of the health apps attempt to provide personalized and tailored information, currently these apps do not have adequate input from users with their personal information to provide tailored feedback. Users need to trust the apps to allow access to enough personal information in order to have more granularly tailored information and feedback. Future research is needed regarding the tension between user’s desire for personalization and privacy protection. Users liked tracking, and particularly reliable automatic tracking, as it required little time and effort from the users. Automatic and accurate sensing of users’ behaviors and states was among one of the top desirable features. Users tended not to want to share personal information due to privacy or security concerns. However, they would share such information if they had control over with whom they shared or could share only with a small group of individuals. Various barriers to adoption as well as motivators for continued use were identified. It is also necessary to mention, both the cost of the app and tangible rewards were identified by participants, indicating the importance of monetary factors. Besides tracking and personalized information, goal setting and reminders were also perceived to be very helpful behavior change features in the health apps.

### Mapping Themes to Theories

After further examining the themes identified via inductive thematic analysis, we found that many of the themes could be mapped to existing technology adoption and behavior change theories. For instance, time and effort requirement for app adoption and continued use is related to the effort expectancy construct in [22]: participants did not use health apps because they did not expect health apps to be simple and easy to use. Lack of app literacy is related to the facilitating conditions construct in [22]: participants did not use health apps because they lacked existing knowledge or guidance of using health apps. The feature liked by the participants, tracking for awareness, ties to the self-observation process of self-regulation in social cognitive theory [21]. Table 2 presents an overview of how some of the identified themes and sub-themes can be applied to different theoretical constructs. We do acknowledge that this list by no means is exhaustive, and additional theories can potentially be mapped to identified themes. The theories included here are frequently used in the context of technology acceptance and behavior change. We also emphasize the added value of connecting our study results to theoretical concepts with the aim of providing theoretical guidance for researchers who intend to examine or develop mobile health technologies and their features.

### Practical Implications

In the United States, only 62% of smartphone owners have used their phone to look up information about a health condition in 2015 [17] and only 19% of smartphone owners have a health app in 2012 [25]. It is possible that the low penetration rate of health apps might result from lack of awareness, just like many of our participants did not realize that they could use their phones to track food and exercise or remind them about their medication. To overcome adoption barriers, first of all, we need to increase awareness of the existence of health apps among the general public. One way to achieve this is through entertainment education—incorporating health app use among characters in popular TV shows might be one way to raise awareness [26]. Primary care doctors may be another important source to recommend health apps to clients. This addresses both the issue of users not knowing which app to choose and the credibility concern. However, to achieve this, health providers themselves should be aware and receptive to health apps. Currently, there is limited research on health providers’ perceptions of health apps and future research regarding this is needed. As for the barrier of not knowing how to use health apps, one possible solution is to provide facilitating conditions such as community technology literacy workshops to help the non-digital native. However, what is more important actually falls on app designers. A well-designed app needs to be intuitive to use without involving users checking a manual. The participants found that many of the existing apps have too many complicated features or options that an average user might not use. A layer design that caters to both experienced and novice users are needed. This also addresses the third barrier—time and effort requirement. Designing an app, which is easy to use and reduces the time and effort put in by the users, is crucial. Thorough usability testing is necessary before app release [27]. The last barrier identified by our participants was cost. Most participants indicated that they only used free apps. In fact, many health apps are free. Existing content analyses indicated that most of the paid apps cost no more than 3 dollars [10]. The paid apps must have features that were not available in any free apps in order to convince the users that it had the price value for them to pay.

The participants also discussed other factors that might motivate them to adopt and continue to use health apps, including social influence and social competition, intangible and tangible rewards, and entertainment and hedonic factors. Participants considered hedonic motivation to be more important for children or the younger generation than for themselves. Friends or family members’ recommendation of health apps was identified as an important factor to increase credibility of the app, thereby describing the importance of social influence. Therefore, designer of health apps should include features publicly demonstrating usage to a user’s close social network to promote wide usage.

Two other prominent features liked or desired by the participants were personalized guidance and reminders. The participants did not just want generic information from the apps; they expected to obtain individualized information from the health apps. Tailoring has been shown to be effective for health communication and education [28, 29]. Health apps have the ability to capture ample user information either through self-input or through automatic sensors. However, many of the existing apps only tailor or personalize minimally. Future research should examine how we can take advantage of smartphones and health apps to capture granular individual information and provide smartly customized guidance and feedback to users [23]. In fact, the reminder feature can also be personalized. Future research should examine whether a personalized reminder can enable users to receive it at the most appropriate time and context to have the maximum effect.

## Conclusions

The current study attempts to expand the limited research on the user perspectives of health apps by conducting a qualitative research with a diverse pool of participants. Although we successfully recruited participants of various age groups, we only recruited five participants outside the university setting. Additionally, due to the constrained working hours, these five participants could not be included into any focus groups and were interviewed individually. Although both the focus group moderator and the interviewer followed a pre-determined guide to ask questions and conduct the qualitative research, the two modes of data collection could introduce biases. For instance, the method of focus groups might lead to participants’ ideas bouncing off of each other and thus growing. At the same time, people might be reluctant to share their perspectives in a group setting of focus groups if they fear that others might not agree with them.

Additionally, despite our effort to recruit a gender balanced pool, about 2/3 of the participants were female. Third, not all the participants had used health apps and the discussion was based on their perception of the examples provided by the researchers in the trigger materials. Their perception might be different when they actually use the apps. Nevertheless, including non-users of health apps, was a deliberate move in order to get insights into the perceptions of those who didn’t use health apps. These insights add an additional layer to the quality of findings. Furthermore, it removes the potential for a positive bias created by a dataset populated only by users (effectively ignoring the non-users). Finally, none of our participants had experience using medical apps that connect to medical devices [30] or for provider communication or medical education [2]. Therefore, the results of this study cannot be apply to medical apps defined by the U. S Food and Drug Administration [30] or Boulos et al. [2]. Despite the limitations, however, our research adds important qualitative evidence to the current research of mHealth through the perspective of a diverse pool of users beyond the young, highly educated group, which can provide important insight for both researchers and app designers to develop and deploy health apps for behavior change intervention. Interpreting the findings based on the theoretical frameworks, we recommend that future research on mHealth and app-based behavior change should focus on developing theory-guided, ease to use, personalized apps with social media and information sharing features that the users can customize and control as well as incentives to foster continued use.

## Additional files

Additional file 1: Consolidated criteria for reporting qualitative studies (COREQ): 32-item checklist. (DOCX 19 kb)
Additional file 2: Focus group and interview guide. (PDF 75 kb)
Additional file 3: Trigger materials. (PDF 873 kb)

## Abbreviations

FCS: Female college student
FG: Focus group
FNS: Female, non-student
MCS: Male college student
MNS: Male, non-student
UTAUT2: Extended unified theory of acceptance and usage of technology

## References

[1] Becker S, Miron-Shatz T, Schumacher N, Krocza J, Diamantidis C, Albrecht U-V (2014). mHealth 2.0: Experiences, possibilities, and perspectives. JMIR mHealth uHealth.

[2] Boulos MN, Brewer AC, Karimkhani C, Buller DB, Dellavalle RP (2014). Mobile medical and health apps: state of the art, concerns, regulatory control and certification. Online J Public Health Inform.

[3] Cafazzo JA, Casselman M, Hamming N, Katzman DK, Palmert MR (2012). Design of an mHealth app for the self-management of adolescent type 1 diabetes: a pilot study. J Med Internet Res.

[4] Kirwan M, Vandelanotte C, Fenning A, Duncan MJ (2013). Diabetes self-management smartphone application for adults with Type 1 diabetes: Randomized controlled trial. J Med Internet Res.

[5] Stinson JN, Jibb LA, Nguyen C, Nathan PC, Maloney AM, Dupuis LL (2013). Development and testing of a multidimensional iPhone pain assessment application for adolescents with cancer. J Med Internet Res.

[6] Carter MC, Burley VJ, Nykjaer C, Cade JE (2013). Adherence to a smartphone application for weight loss compared to website and paper diary: pilot randomized controlled trial. J Med Internet Res.

[7] Turner-McGrievy GM, Beets MW, Moore JB, Kaczynski AT, Barr-Anderson DJ, Tate DF (2013). Comparison of traditional versus mobile app self-monitoring of physical activity and dietary intake among overweight adults participating in an mHealth weight loss program. J Am Med Inform Assoc.

[8] Abroms LC, Westmaas JL, Bontemps-Jones J, Ramani R, Mellerson J (2013). A content analysis of popular smartphone apps for smoking cessation. Am J Prev Med.

[9] Breton ER, Fuemmeler BF, Abroms LC (2011). Weight loss—there is an app for that! But does it adhere to evidence-informed practices?. Transl Behav Med.

[10] Cowan LT, Van Wagenen SA, Brown BA, Hedin RJ, Seino-Stephan Y, Hall PC (2013). Apps of steel: Are exercise apps providing consumers with realistic expectations? A content analysis of exercise apps for presence of behavior change theory. Health Educ Behav.

[11] Direito A, Dale LP, Shields E, Dobson R, Whittaker R, Maddison R (2014). Do physical activity and dietary smartphone applications incorporate evidence-based behaviour change techniques?. BMC Public Health.

[12] Middelweerd A, Mollee JS, van der Wal CN, Brug J, te Velde SJ (2014). Apps to promote physical activity among adults: a review and content analysis. Int J Behav Nutr Phys Act.

[13] Azar KMJ, Lesser LI, Laing BY, Stephens J, Aurora MS, Burke LE (2013). Mobile applications for weight management theory-based content analysis. Am J Prev Med.

[14] Zahry NR, Cheng Y, Peng W. Content analysis of diet-related mobile apps: A self-regulation perspective. Health Commun. 2016; doi: 10.1080/10410236.2015.1072123. 10.1080/10410236.2015.1072123 26940817

[15] Grindrod KA, Li M, Gates A (2014). Evaluating user perceptions of mobile medication management applications with older adults: a usability study. JMIR mHealth uHealth.

[16] Dennison L, Morrison L, Conway G, Yardley L (2013). Opportunities and challenges for smartphone applications in supporting health behavior change: Qualitative study. J Med Internet Res.

[17] Smith A. U.S. Smartphone Use in 2015. Pew Research Center. 2015. http://www.pewinternet.org/2015/04/01/us-smartphone-use-in-/. Accessed 30 Aug 2015.

[18] Smartphone ownership penetration in the United Kingdom (UK) in 2012-2015, by age. http://www.statista.com/statistics/271851/smartphone-owners-in-the-united-kingdom-uk-by-age/. Accessed 1 Dec 1 2015.

[19] Klasnja P, Pratt W (2012). Healthcare in the pocket: Mapping the space of mobile-phone health interventions. J Biomed Inform.

[20] Venkatesh V, Thong JY, Xu X (2012). Consumer acceptance and use of information technology: Extending the unified theory of acceptance and use of technology. MIS Q.

[21] Bandura A (1991). Social cognitive theory of self-regulation. Organ Behav Hum Decis Process.

[22] Gould SJ (1990). Health consciousness and health behavior: the application of a new health consciousness scale. Am J Prev Med.

[23] Spruijt-Metz D, Nilsen W (2014). Dynamic models of behavior for just-in-time adaptive interventions. IEEE Pervasive Comput.

[24] Braun V, Clarke V (2006). Using thematic analysis in psychology. Qual Res Psychol.

[25] Fox S, Duggan M. Mobile Health 2012. Pew Research Center. 2012. http://www.pewinternet.org/files/old-media//Files/Reports/2012/PIP_MobileHealth2012_FINAL.pdf. Accessed 30 Aug 2015.

[26] Singhal A, Cody MJ, Rogers EM, Sabido M (2003). Entertainment-education and social change: History, research, and practice.

[27] Zapata BC, Fernández-Alemán JL, Idri A, Toval A (2015). Empirical studies on usability of mHealth apps: A systematic literature review. J Med Syst.

[28] Kreuter MW, Oswald DL, Bull FC, Clark EM (2000). Are tailored health education materials always more effective than non-tailored materials?. Health Educ Res.

[29] Noar SM, Benac CN, Harris MS (2007). Does tailoring matter? Meta-analytic review of tailored print health behavior change interventions. Psychol Bull.

[30] U. S. Food and Drug Administration. Mobile medical applications: Guidance for industry and food and drug administration staff. http://www.fda.gov/downloads/MedicalDevices/…/UCM263366.pdf Accessed 28 July 2016.

[31] Davis V (1989). Perceived usefulness, perceived ease of use, and user acceptance of information technology. MIS Q.

[32] Fishbein M, Ajzen I (1975). Belief, attitude, intention, and behavior: An introduction to theory and research.

[33] Ajzen I (1991). The theory of planned behavior. Organ Behav Hum Decis Process.

[34] Rogers EM (1995). Diffusion of innovations.

[35] Baumeister RF, Vohs KD, Tice DM (2007). The strength model of self-control. Curr Dir Psychol Sci.

[36] Locke EA, Latham GP (1990). A theory of goal setting and task performance.

[37] Becker MH, Radius SM, Rosenstock IM (1978). Compliance with a medical regimen for asthma: A test of the health belief model. Public Health Rep.

