# Supplementary material for: A qualitative study of user perceptions of mobile health apps
Source: BMC Public Health. 2016 Nov 14;16:1158. doi: 10.1186/s12889-016-3808-0 (PMC5109835; doi:10.1186/s12889-016-3808-0)
Supplement: Additional file 1: — Consolidated criteria for reporting qualitative studies (COREQ): 32-item checklist. (DOCX 19 kb) [file 12889_2016_3808_MOESM1_ESM.docx]

**Additional file 1. Consolidated criteria for reporting qualitative studies (COREQ): 32-item checklist**

| **No. Item** | **Guide questions/description** | **Reported on Manuscript** |
| --- | --- | --- |
| **Domain 1: Research team and reﬂexivity** |  |  |
| *Personal Characteristics* |  |  |
| 1. Interviewer/facilitator | Which author/s conducted the inter view or focus group? | In focus groups, the second author served as the moderator and the third author recorded the sessions and took notes. The fourth author conducted the interviews. |
| 2. Credentials | What were the researcher’s credentials? E.g. PhD, MD | The moderator and interviewer held MA. |
| 3. Occupation | What was their occupation at the time of the study? | The interviewer and the moderator were doctoral students. |
| 4. Gender | Was the researcher male or female? | The moderator was female and the interviewer was male. |
| 5. Experience and training | What experience or training did the researcher have? | Academic training and past experience in qualitative research methods. |
| ***Relationship with participants*** |  |  |
| 6. Relationship established | Was a relationship established prior to study commencement? | No prior relationship. |
| 7. Participant knowledge of the interviewer | What did the participants know about the researcher? E.g., personal goals, reasons for doing the research | No prior knowledge of the interviewer. |
| 8. Interviewer characteristics | What characteristics were reported about the interviewer/facilitator? E.g., Bias, assumptions, reasons and interests in the research topic | All the authors had a positive attitude towards health apps, but the authors strived to remain neutral in the conversations with participants. |
| **Domain 2: study design** |  |  |
| *Theoretical framework* |  |  |
| 9. Methodological orientation and Theory | What methodological orientation was stated to underpin the study? e.g. grounded theory, discourse analysis, ethnography, phenomenology, content analysis | Inductive Thematic Analysis. |
| *Participant selection* |  |  |
| 10. Sampling | How were participants selected? e.g. purposive, convenience, consecutive, snowball | Purposive sampling. |
| 11. Method of approach | How were participants approached? e.g. face-to-face, telephone, mail, email | Focus group participants were recruited via email and a subject pool system. For interviews, participants were recruited through in-person interaction. |
| 12. Sample size | How many participants were in the study? | 44 participants. |
| 13. Non-participation | How many people refused to participate or dropped out? Reasons? | All participants who completed the consent form completed the study. |
| *Setting* |  |  |
| 14. Setting of data collection | Where was the data collected? e.g. home, clinic, workplace | The focus groups were conducted in a conference room. The interviews were conducted at participants’ workplace, home or a nearby café. |
| 15. Presence of non-participants | Was anyone else present besides the participants and researchers? | Only the researchers were present during data collection with the participants. |
| 16. Description of sample | What are the important characteristics of the sample? e.g. demographic data, date | The details about study sample are provided in Table 1 |
| *Data collection* |  |  |
| 17. Interview guide | Were questions, prompts, guides provided by the authors? Was it pilot tested? | The research team developed the questions, prompts, and guides jointly. |
| 18. Repeat interviews | Were repeat interviews carried out? If yes, how many? | No. |
| 19. Audio/visual recording | Did the research use audio or visual recording to collect the data? | All focus groups and interviews were audio recorded. |
| 20. Field notes | Were ﬁeld notes made during and/or after the interview or focus group? | Field notes were made during the focus group. |
| 21. Duration | What was the duration of the inter views or focus group? | 40-90 minutes for focus groups and 30-45 minutes for interviews. |
| 22. Data saturation | Was data saturation discussed? | Recruitment was not based on data saturation. |
| 23. Transcripts returned | Were transcripts returned to participants for comment and/or correction? | No. |
| **Domain 3: analysis and ﬁndings** |  |  |
| *Data analysis* |  |  |
| 24. Number of data coders | How many data coders coded the data? | Three authors coded the data and each transcript was coded by two authors separately. |
| 25. Description of the coding tree | Did authors provide a description of the coding tree? | No. |
| 26. Derivation of themes | Were themes identiﬁed in advance or derived from the data? | Themes were derived from the data. |
| 27. Software | What software, if applicable, was used to manage the data? | NVivo. |
| 28. Participant checking | Did participants provide feedback on the ﬁndings? | No. |
| *Reporting* |  |  |
| 29. Quotations presented | Were participant quotations presented to illustrate the themes/ﬁndings? Was each quotation identiﬁed? e.g. participant number | Yes. Each quotation was identified by which focus group the participant was from and the gender of the participant or by the profession of the interviewee. |
| 30. Data and ﬁndings consistent | Was there consistency between the data presented and the ﬁndings? | Yes. |
| 31. Clarity of major themes | Were major themes clearly presented in the ﬁndings? | Major themes are summarized in the results and Table 2. |
| 32. Clarity of minor themes | Is there a description of diverse cases or discussion of minor themes? | Yes, e.g., subthemes of barriers include diverse cases. |

Reference: Tong A, Sainsbury P, Craig J. Consolidated criteria for reporting qualitative research (COREQ): a 32-item checklist for interviews and focus groups. *International Journal for Quality in Health Care*. 2007. Volume 19, Number 6: pp. 349 – 357
