# Supplementary material for: A qualitative study of user perceptions of mobile health apps
Source: BMC Public Health. 2016 Nov 14;16:1158. doi: 10.1186/s12889-016-3808-0 (PMC5109835; doi:10.1186/s12889-016-3808-0)
Supplement: Additional file 2: — Focus group and interview guide. (PDF 75 kb) [file 12889_2016_3808_MOESM2_ESM.pdf]

Appendix 2. Focus group and interview guide

1. Please tell us your first name, age and major (depending on whether participants are students or non-students).
2. Can you tell us about the kinds of mobile phone apps you use?
3. How frequently do you use them?
4. Do you have any health and fitness apps on your phone?
5. How long ago did you download them?
6. How frequently do you use them?
7. What kind of medical/health apps do you think exist?
8. For all of you who do not have any health & fitness phone apps – why don't you?
9. For everyone who uses health & fitness apps--What do you like about the app? What are you favorite features? What do you dislike about the app? Is there anything you would like to change about it? Why did you get (What motivated you to get) the health app? Is there any activity you would not carry out if you did not have your health related app, for example, counting calories, going to the gym, counting foot-steps, etc)? How has using the health mobile app increased your knowledge about health or made you live a more healthy life?? In what ways?

Use the trigger materials (see Appendix 2) to introduce various examples of health apps and their features. For each example, the participants were asked:
10. What do you like about the app? What are your favorite features?
11. What do you dislike about the app? Is there anything you would like to change about it?

After discussing the trigger materials, the following questions were asked.
12. Do (Would) you evaluate the quality and credibility of an app before you download it? How?
13. Did you download a health app on your own or did someone suggest it to you (example, a friend, a doctor, etc)?
14. How does cost of the app influence your decision to download it?
15. If you could improve something about an app, so as to continue using it, what would it be?
16. What would ensure your continued usage of an app?
17. Finally, would you be likely to download health & fitness apps? Why?
