# Supplementary material for: A qualitative study of user perceptions of mobile health apps
Source: BMC Public Health. 2016 Nov 14;16:1158. doi: 10.1186/s12889-016-3808-0 (PMC5109835; doi:10.1186/s12889-016-3808-0)

Trigger Materials

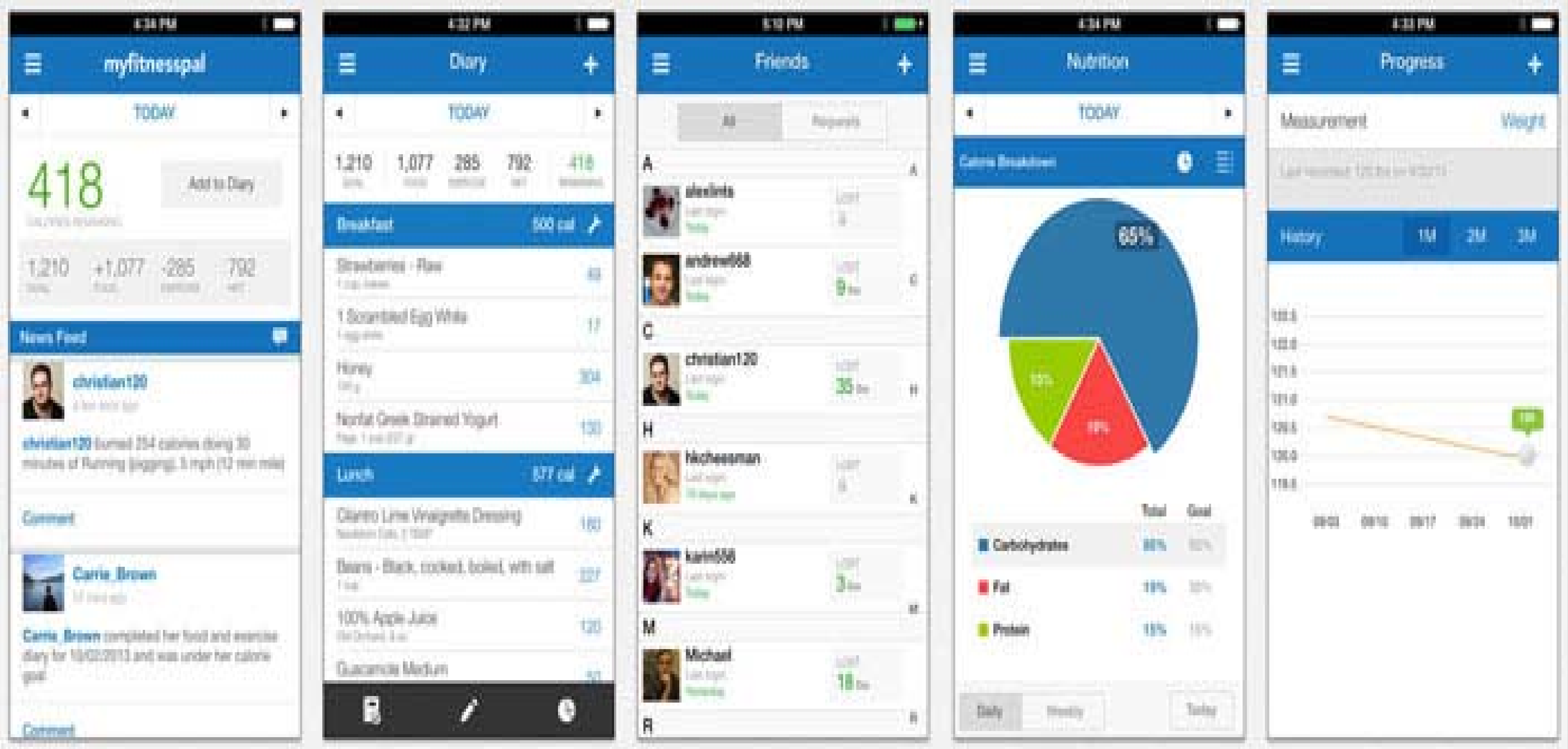

1. Goal setting

- Some apps help users set goals and work toward meeting them.

a. Weight goal

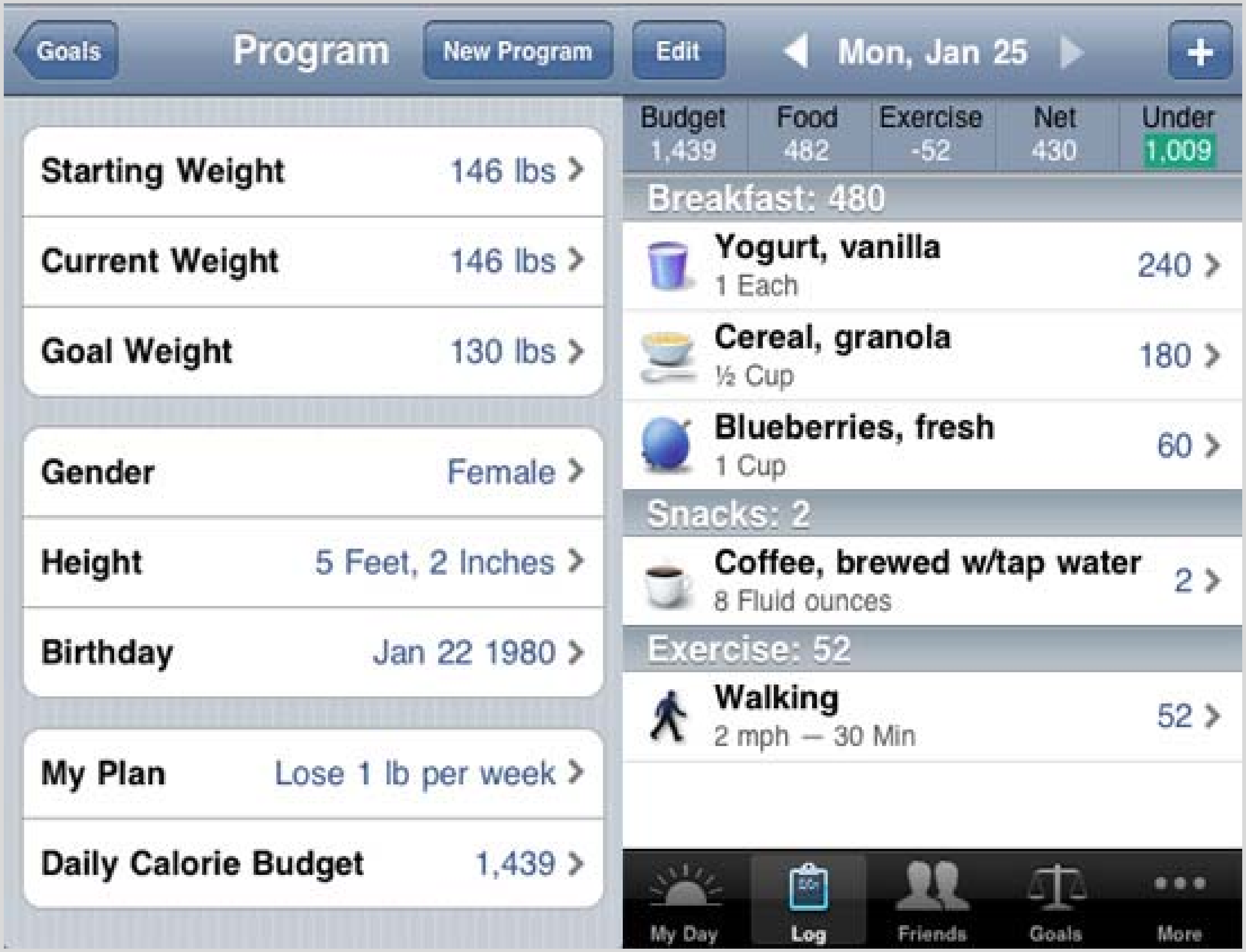

- Helps the user achieve their weight goal by tracking food intake, physical activity, etc.

b. Exercise plan

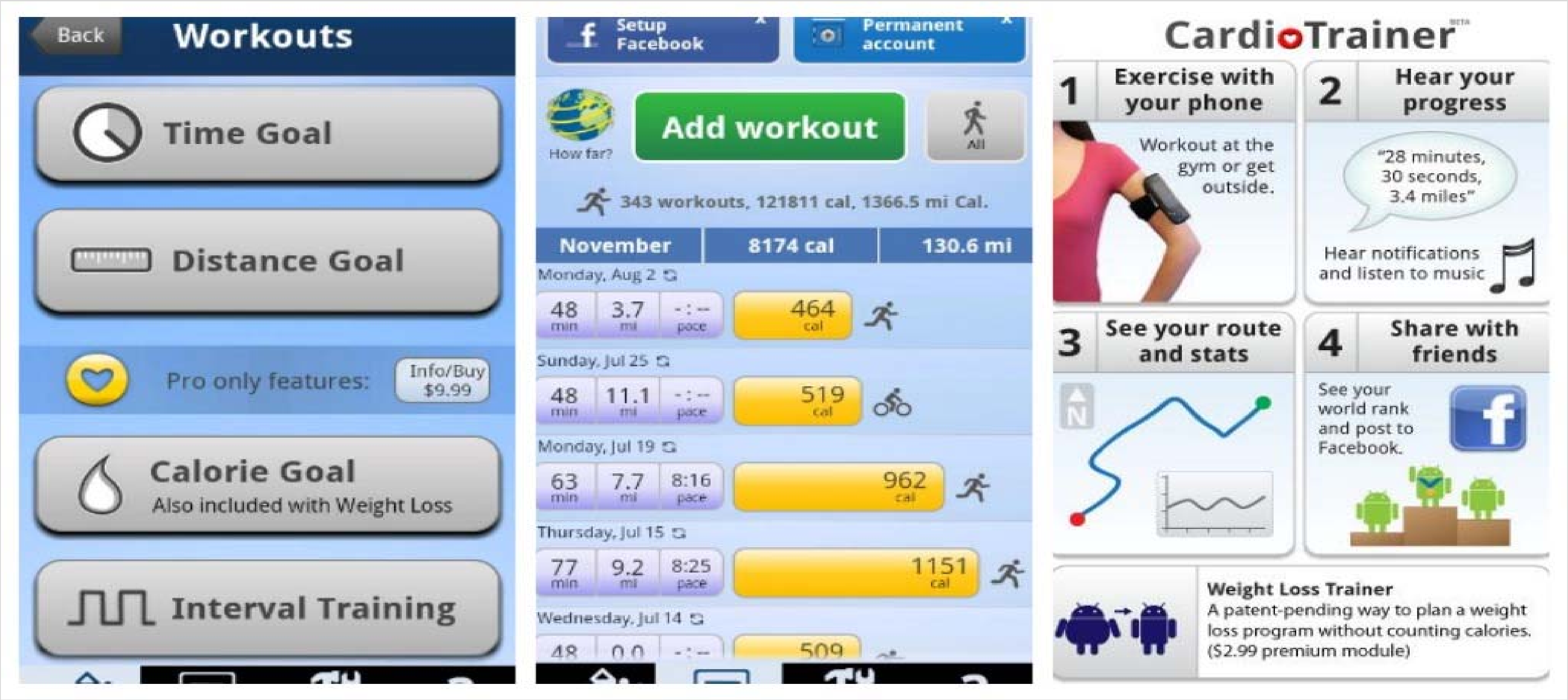

- CardioTrainer, helps the user achieve their health goals by tracking physical activities, setting goals, creating a plan, etc.

2. Behavior monitoring/tracking

- These apps track or monitor the users’ health related behaviors.

a. Food intake monitoring

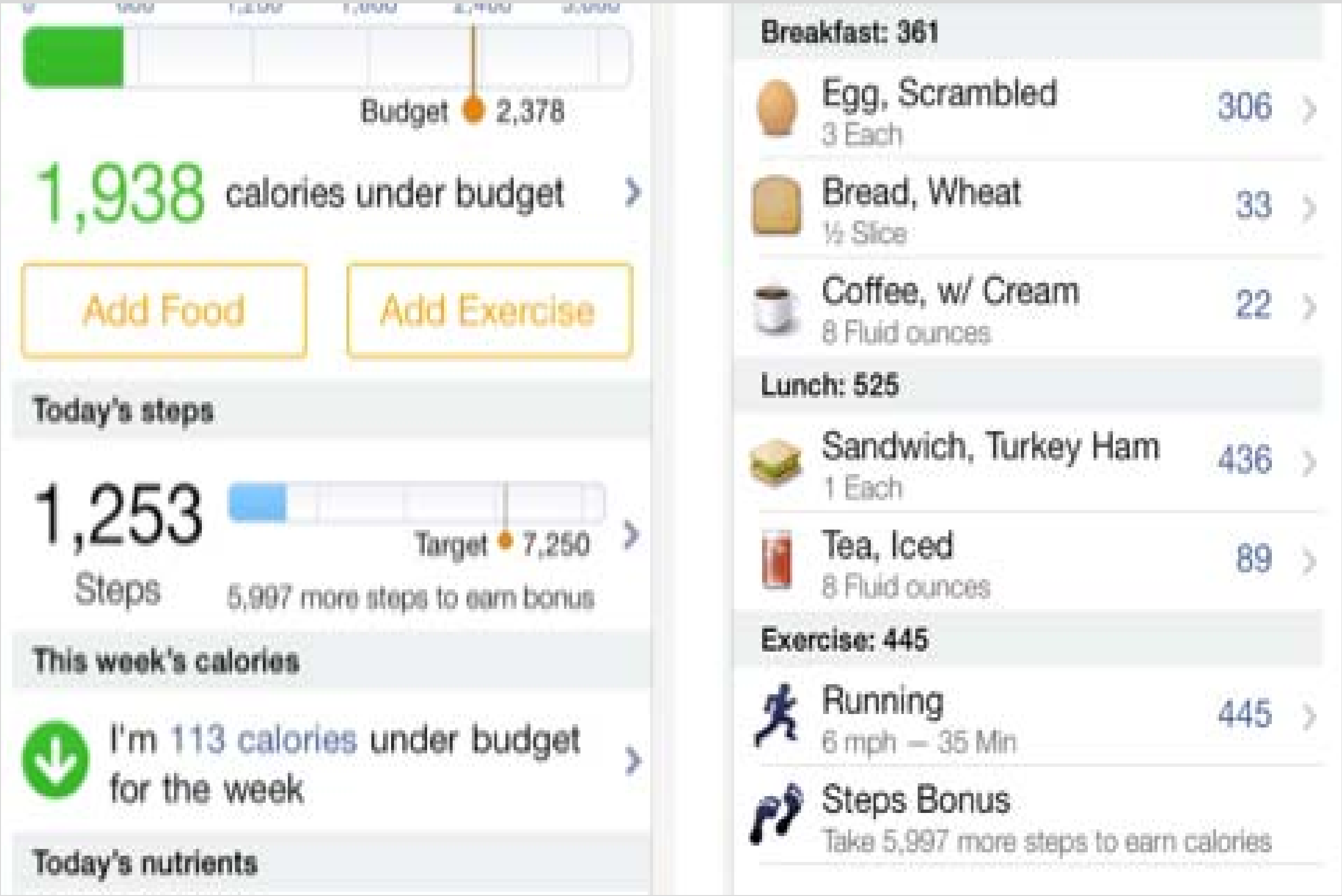

These apps, track the amount and type of food consumed by an individual per day to help with their health.

b. Exercise monitoring

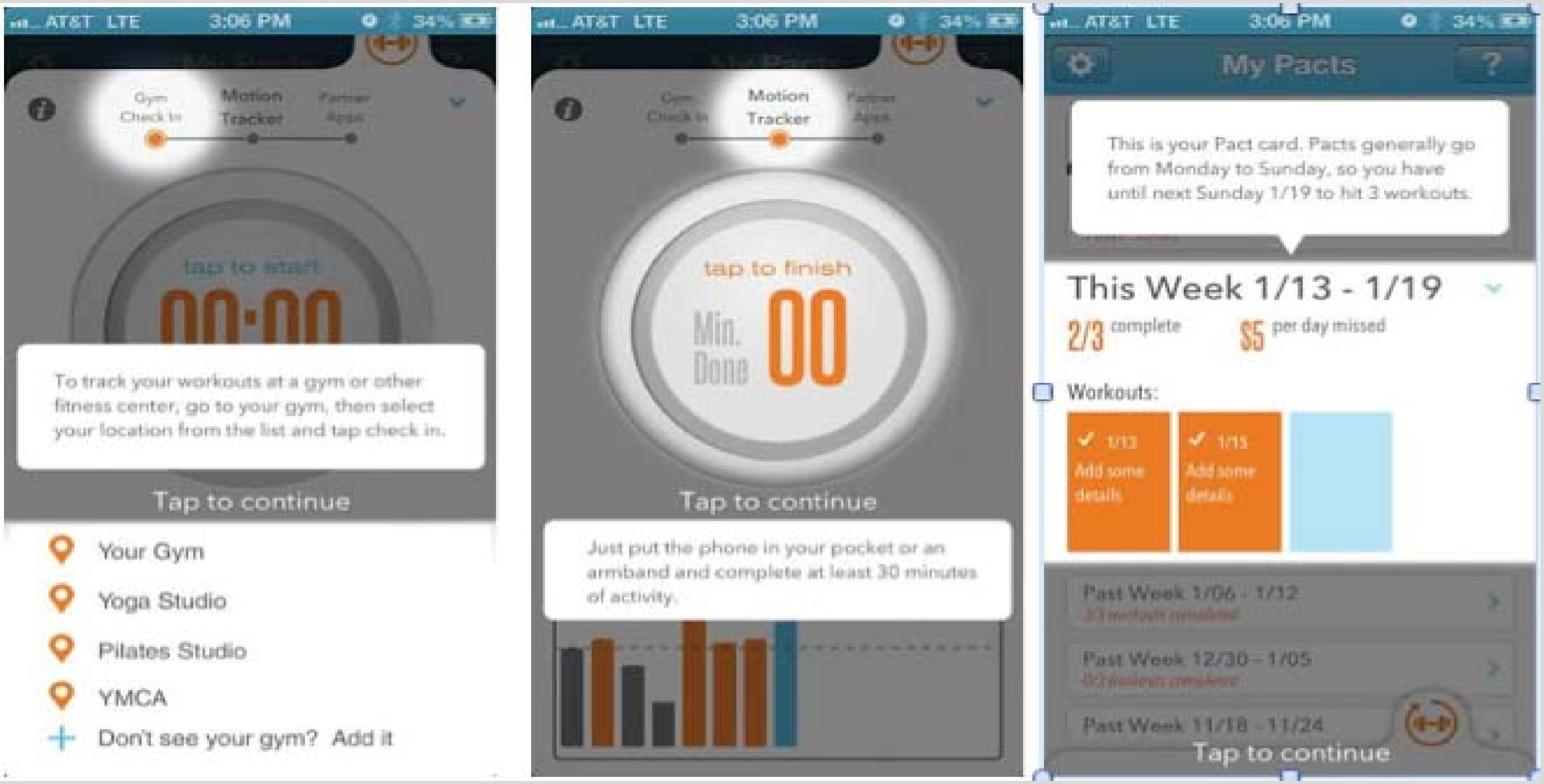

- Apps such as GymPact, track or monitor if you follow the exercise regimen you planned

c. Pedometer

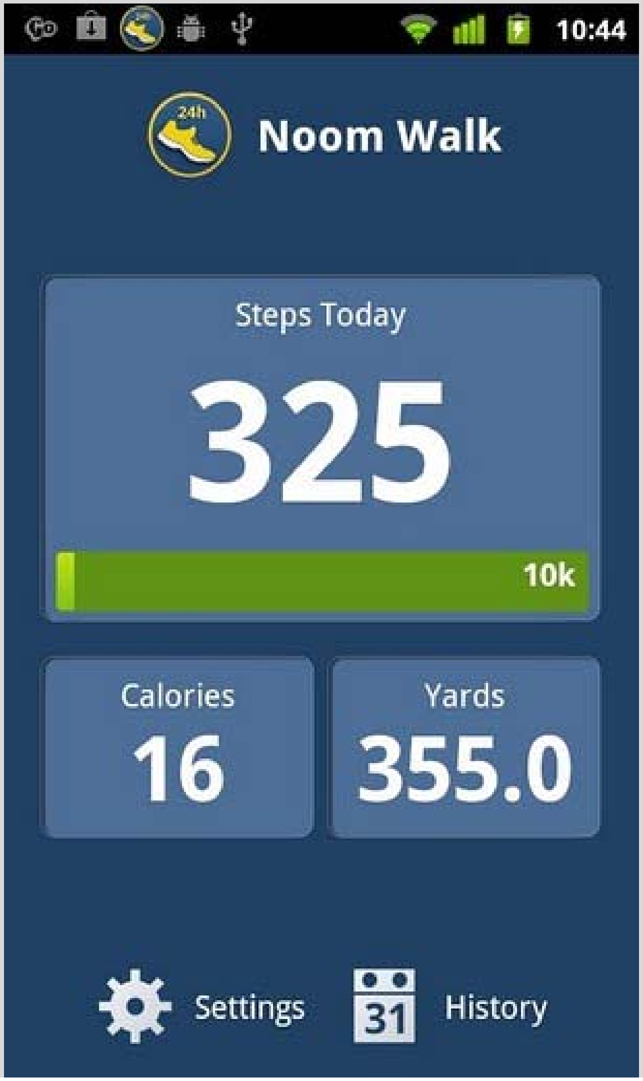

- The smartphone senses and counts the individual's steps and can generate information such as the distance covered by the person and the calories they could've burned due to walking that amount.

d. Use camera (taking photos of food, scan up bar)

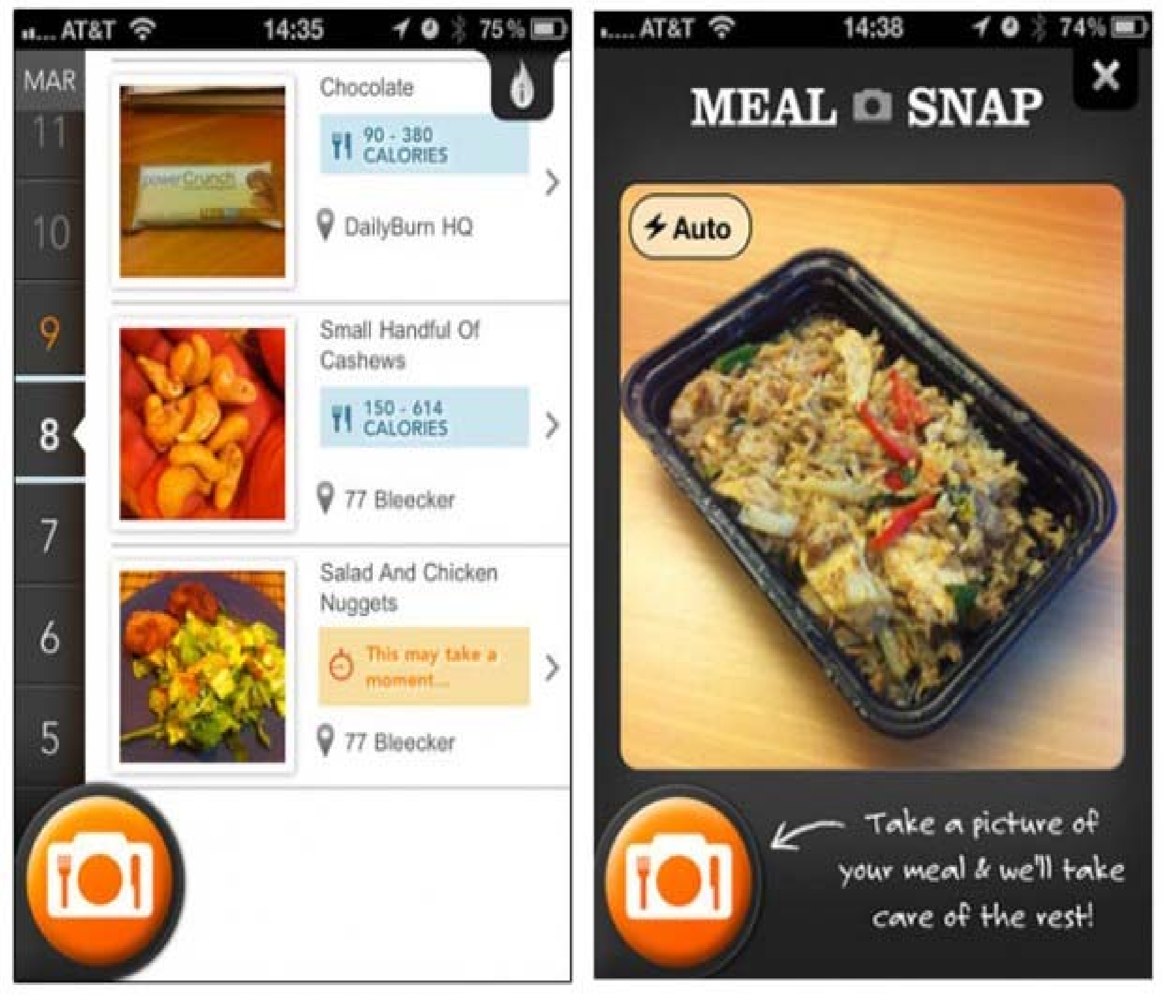

- Meal Snap is a camera utilizing calorie counting app. When a user takes an image and uploads it to the app, the app uses crowdsourcing (through Mechanical Turk) to identify the food and searches for calorie information in a nutritional database.

3. Reminders

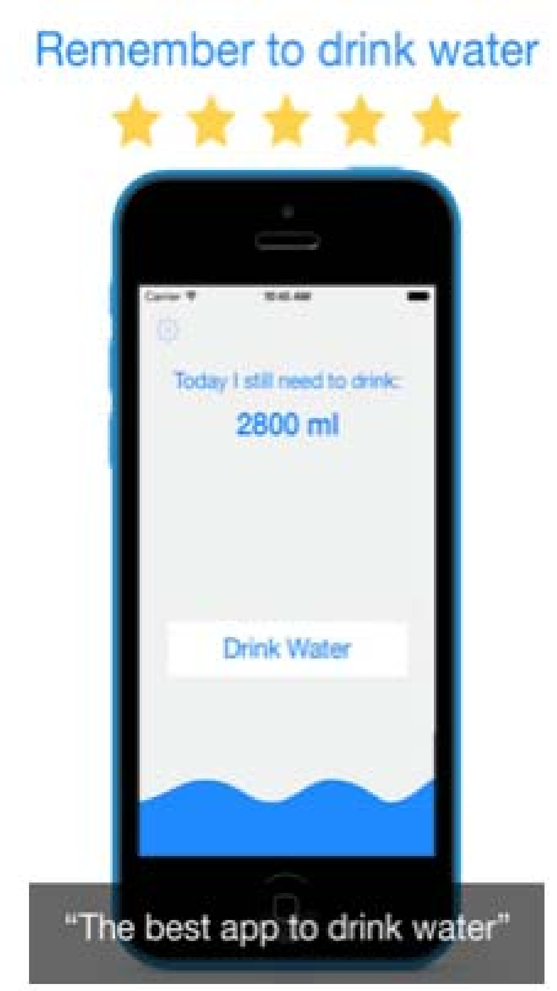

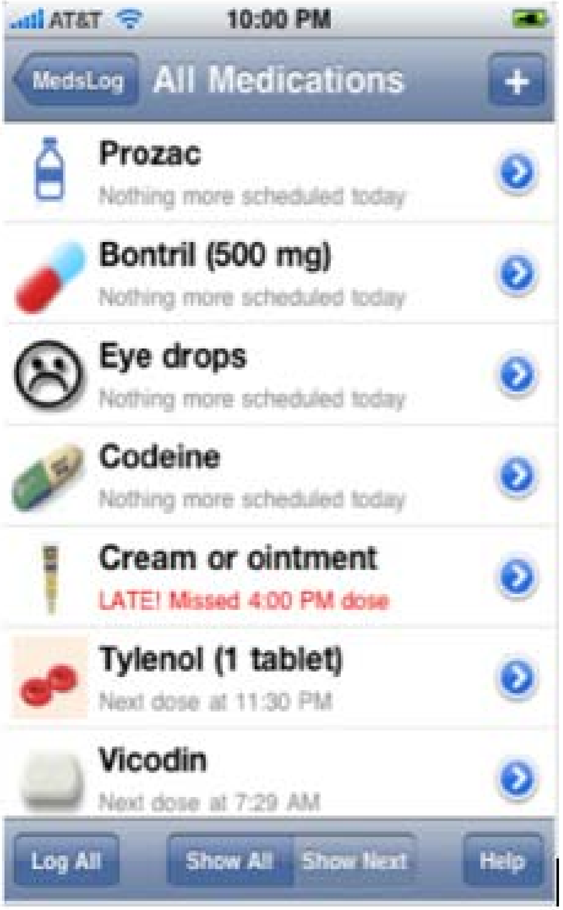

- These apps can provide reminders to users to complete certain tasks, such as taking medication on time, among other things.

4. Progress showing

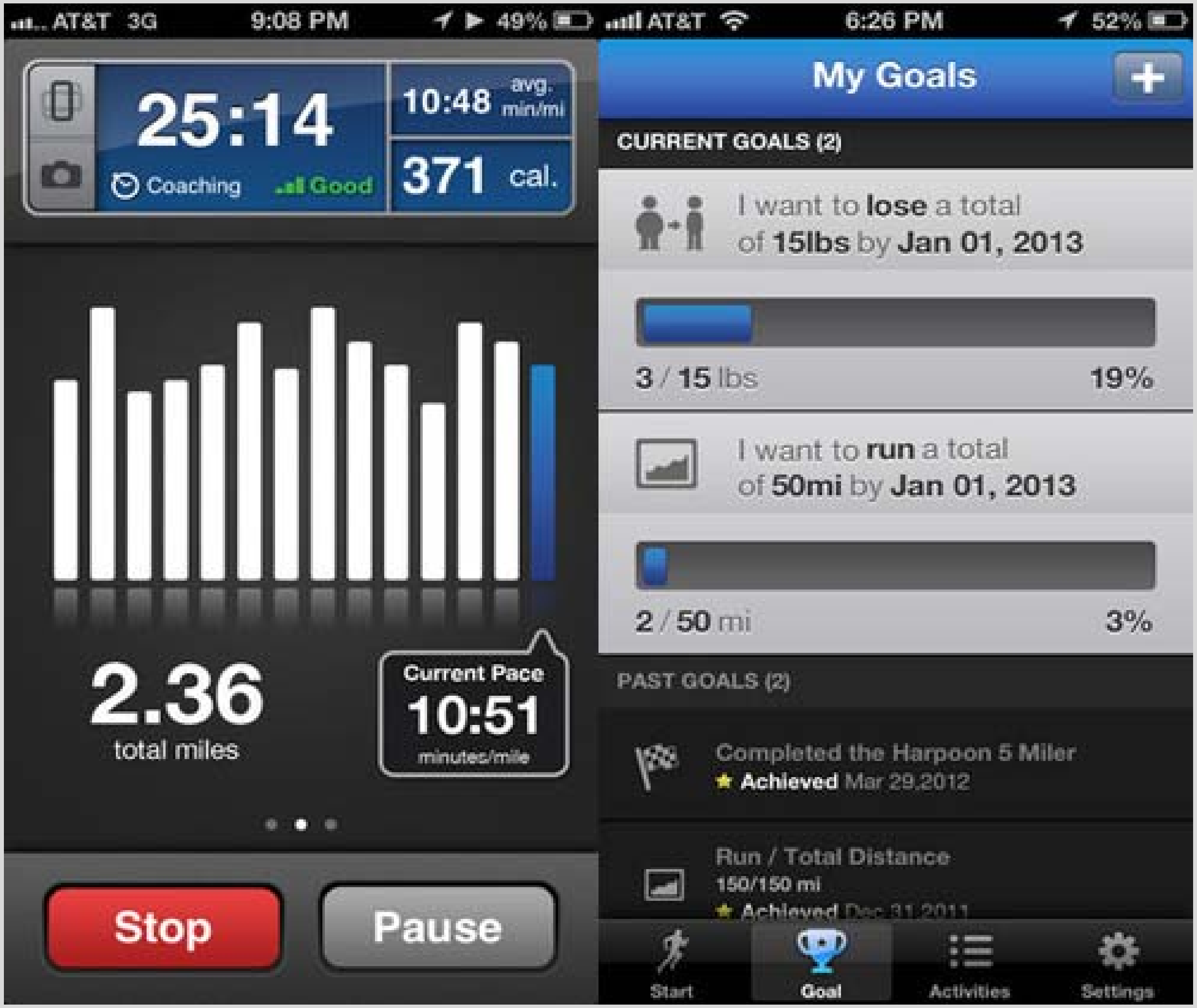

- Progress showing apps, such as RunKeeper, can show an individual their progress with respect to performing a particular task.

5. Social Networking

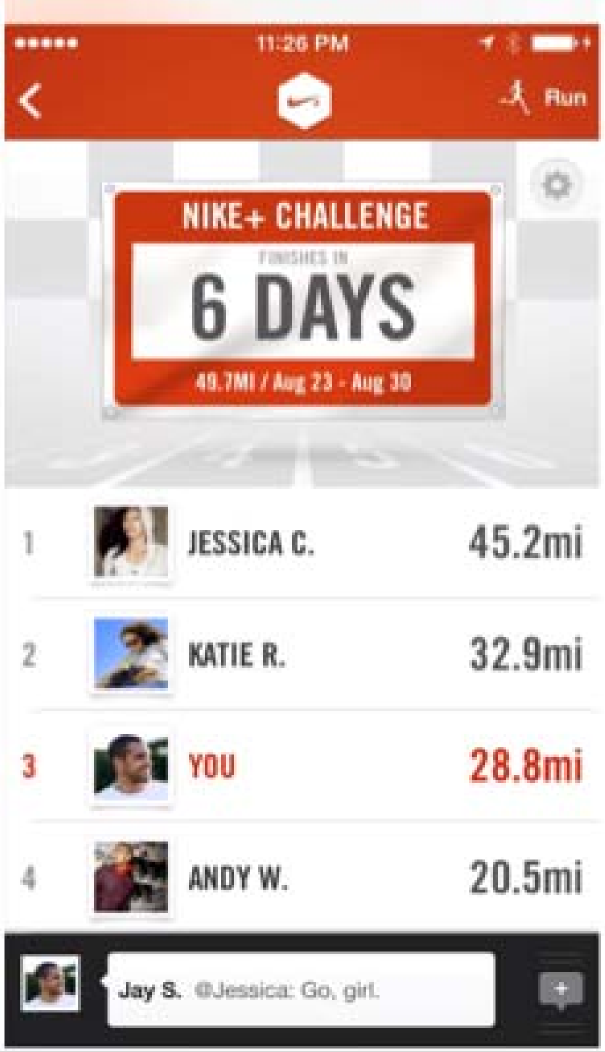

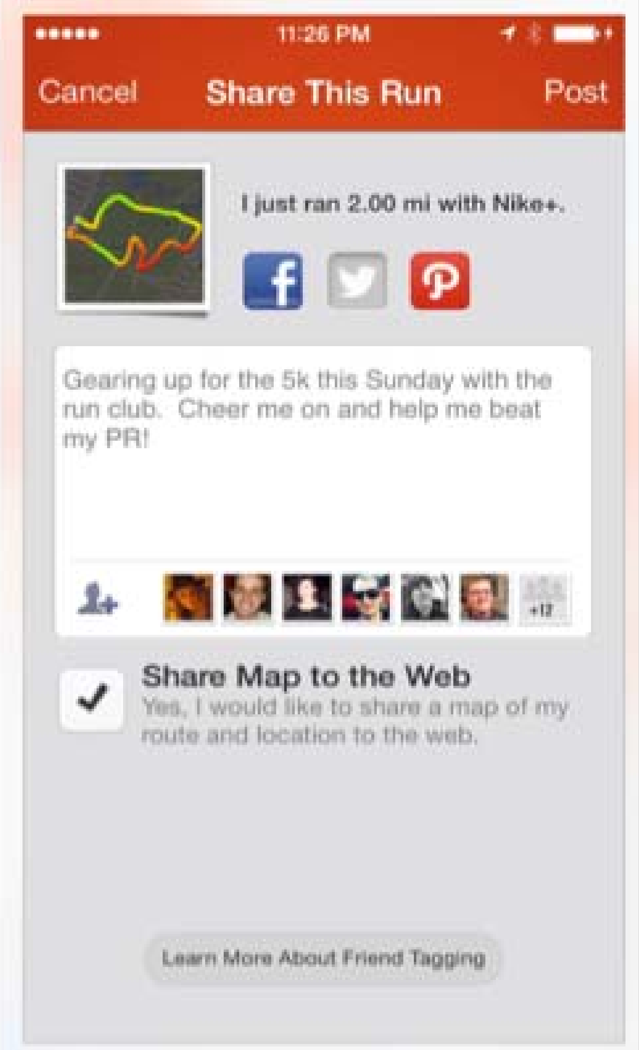

- Most achievements can be shared via social media & even add ‘friends’ to their network.
- Get cheered on: Rally your cheering section. Post the start of your run to Facebook or Path and hear real-time cheers for each like or comment you receive.

5. Social networking

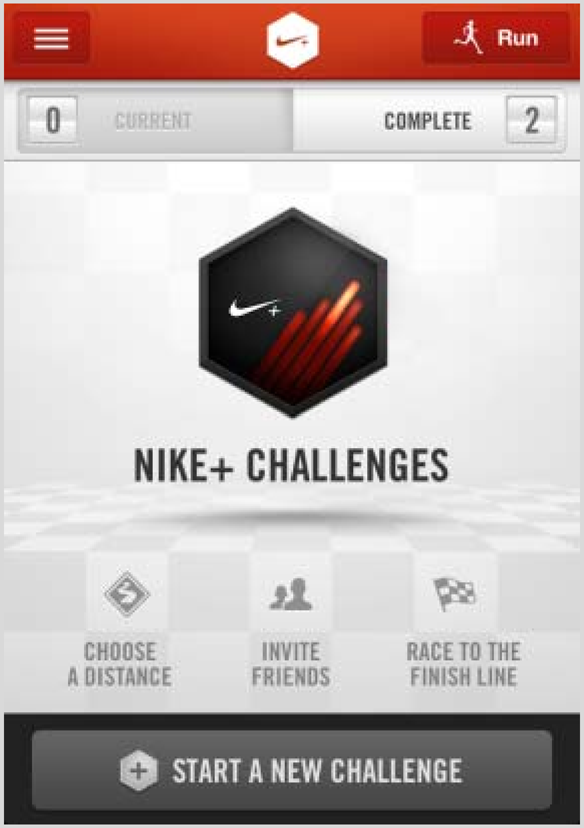

- Hypothetically, would an app where an individual pairs up with another individual to defeat another team in a health related activity work as a successful app?

6. Sensing

- These apps 'sense' different aspects about the users and can therefore help them.

a. Where you are

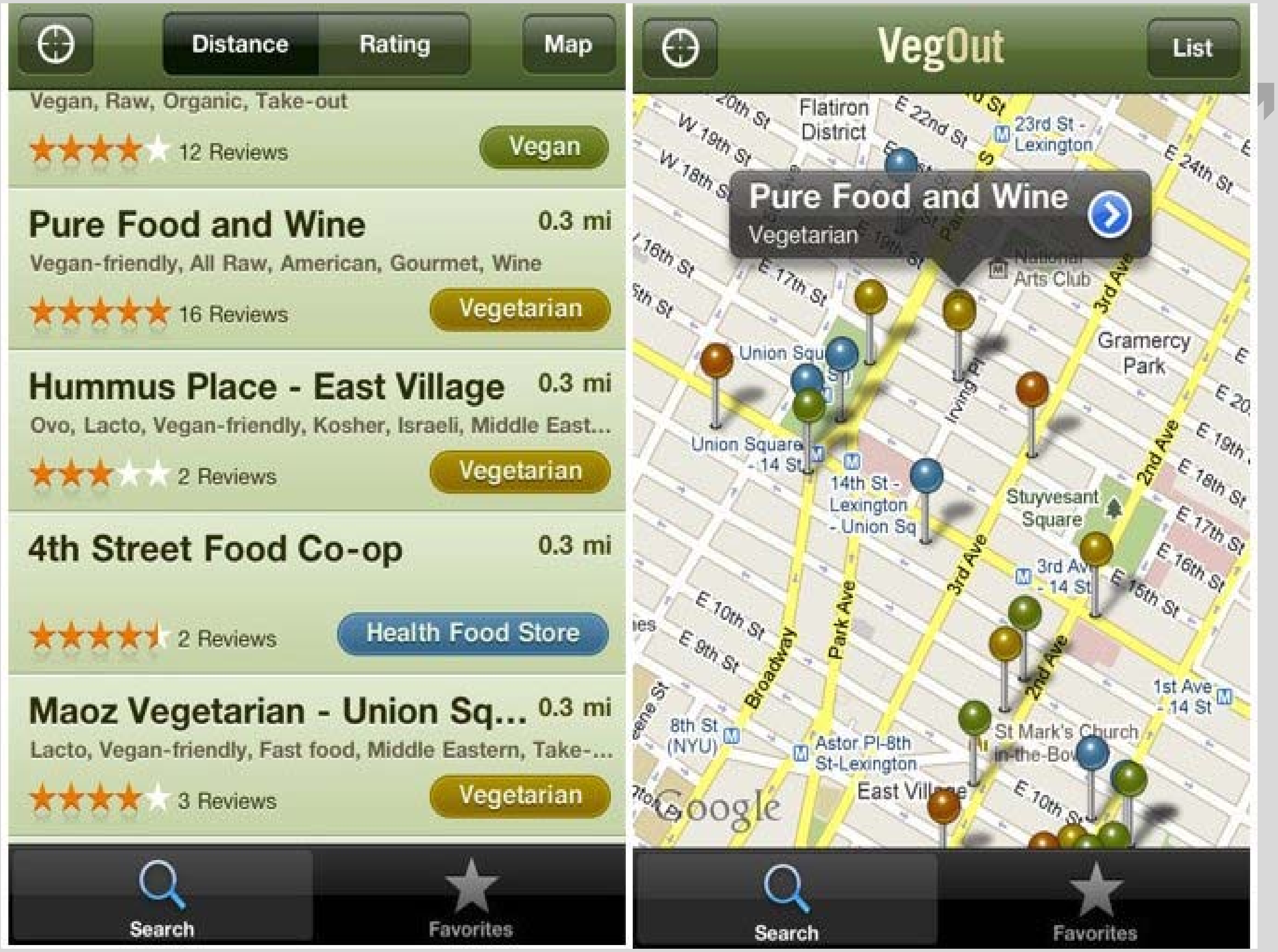

By sensing where the users are, these apps can guide them to healthier food options, instead of fast food, for example.

b. What you're doing

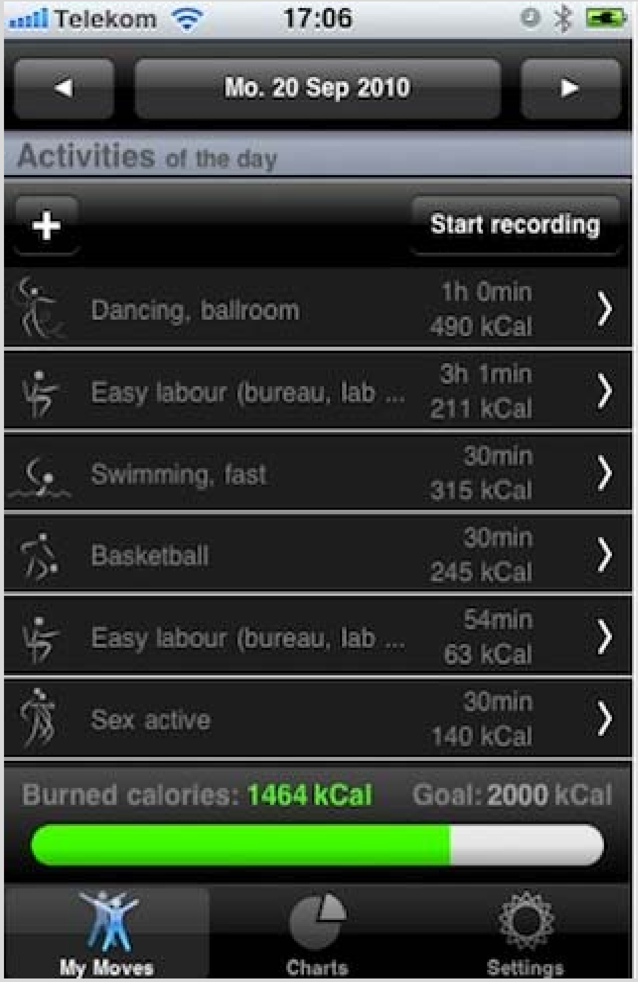

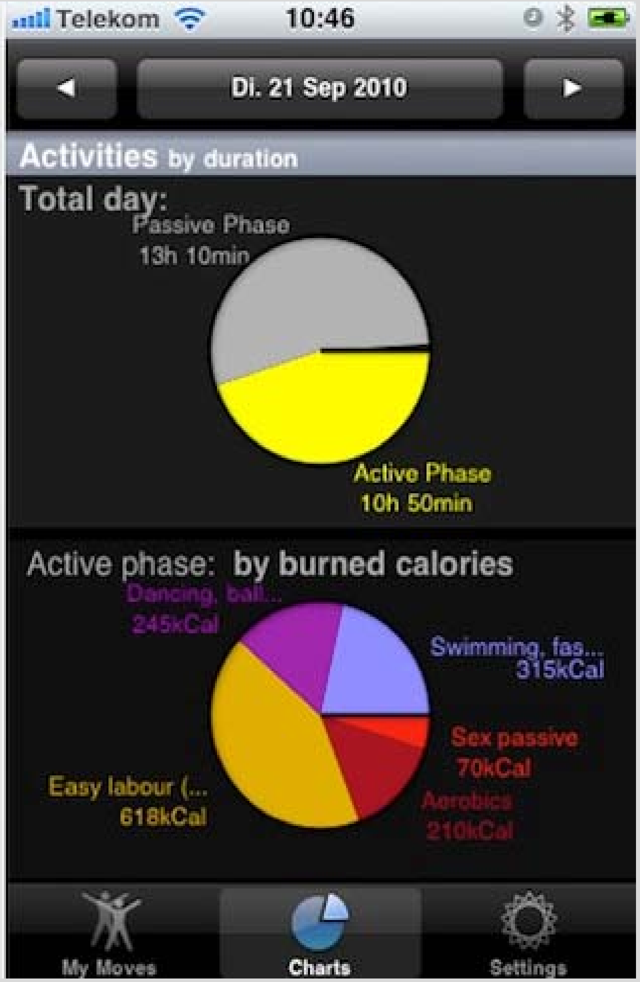

- Apps such as Movator, sense what the individual is doing to document all their activities per day and determine calories burned, the active phases of their day, the passive phases of their day, etc.

- Another app that can be categorized as a sensing app is GymPact mentioned above. It senses when you are at the gym and monitors your exercise.

c. Your mood (who you are with/how you feel)

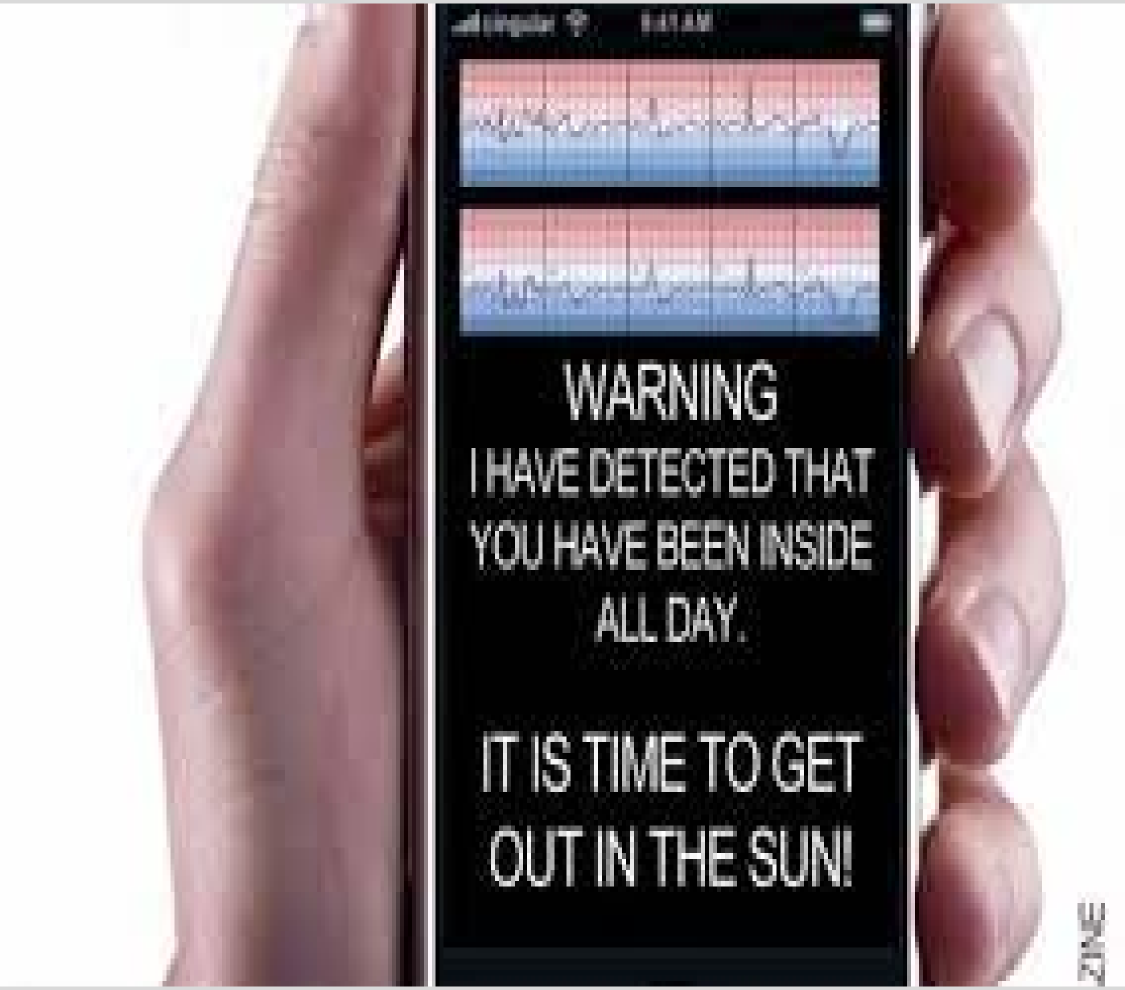

- Apps which can gauge the user's mood exist. Most of these apps are used for entertainment purposes. Would you like an app that sensed your mood and prompted you to take healthy actions, which may improve your life?

7. Using entertainment

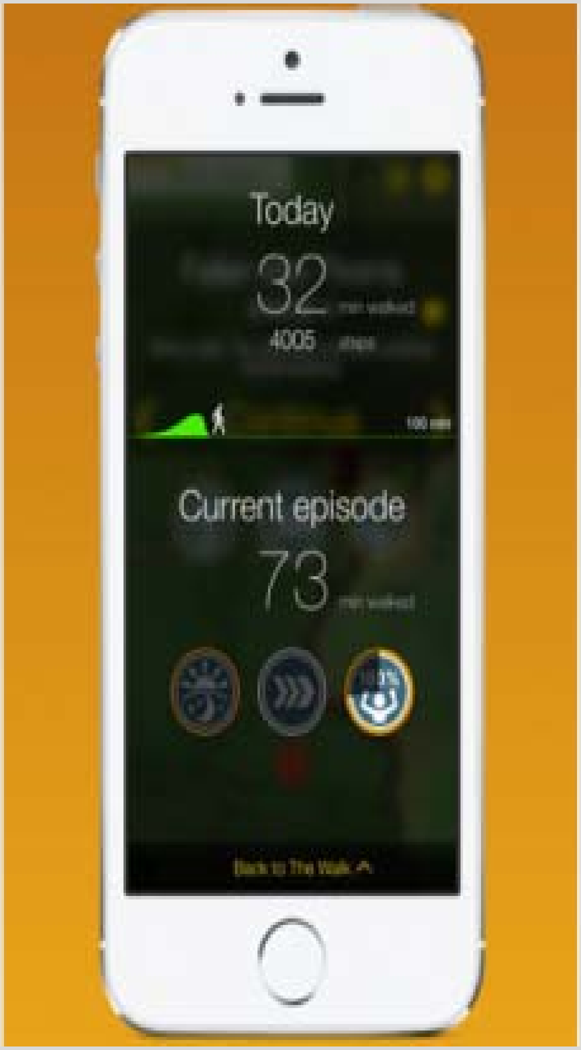

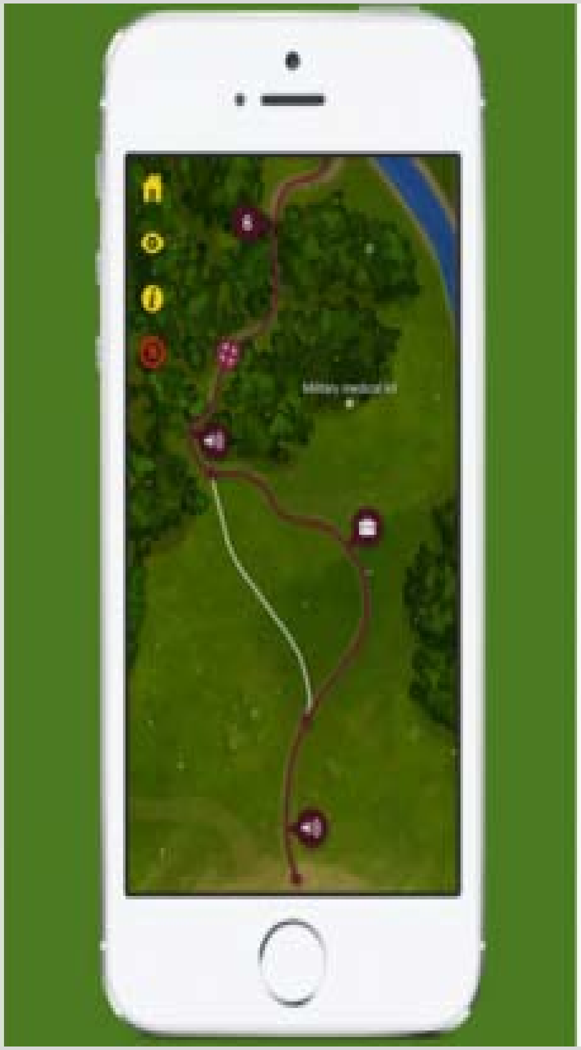

- Entertainment is something users enjoy. This could be in the form of celebrities encouraging users to continue running to reach their goals, video games that sprout health related information for you or act as teaching platforms. Ex: ‘The Walk’ (Users play a game where they avoid zombies who have taken over the world. The app provides the running route and things they must avoid, helping the user obtain physical activity.)

8. Tips/advice giving (increasing the accessibility of health information)

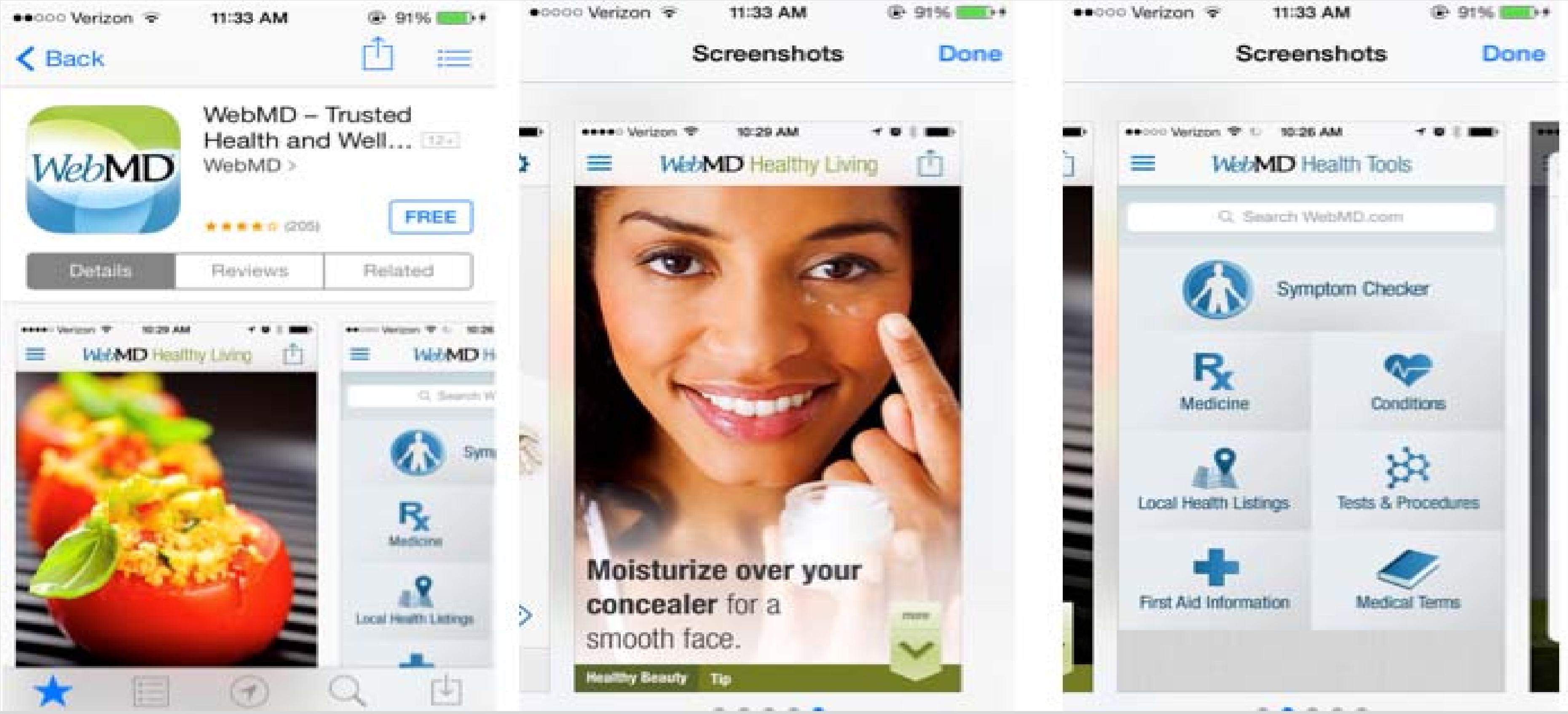

- Some apps help the user by simply providing them with health related information they need or want.

9. Teaching

- Some apps can be used as teaching guides. They can provide ‘how-to’ information or tips to improve the users health related behaviors.

a. Workout

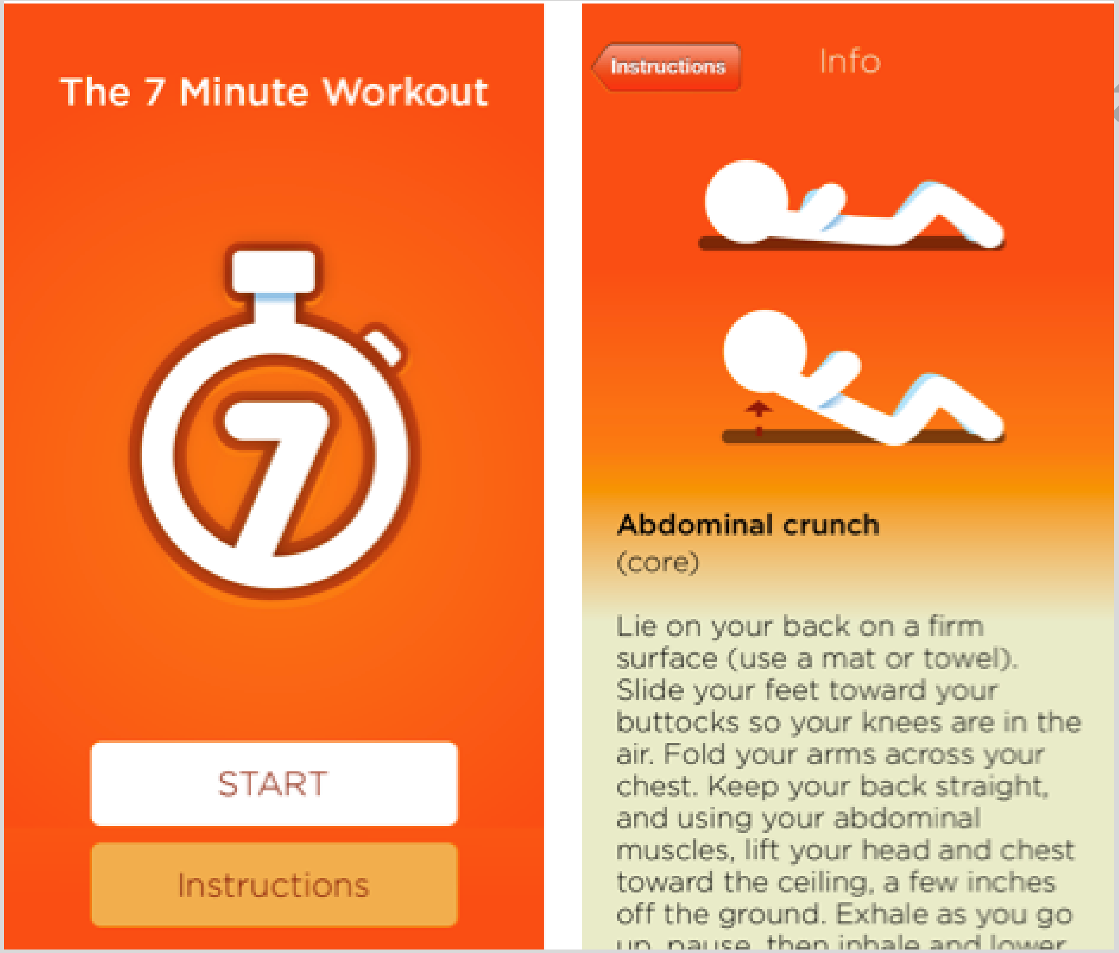

- These apps teach users either quick workout tips or efficient ways to work out so as to reach their health goals.

b. Better teaching habits

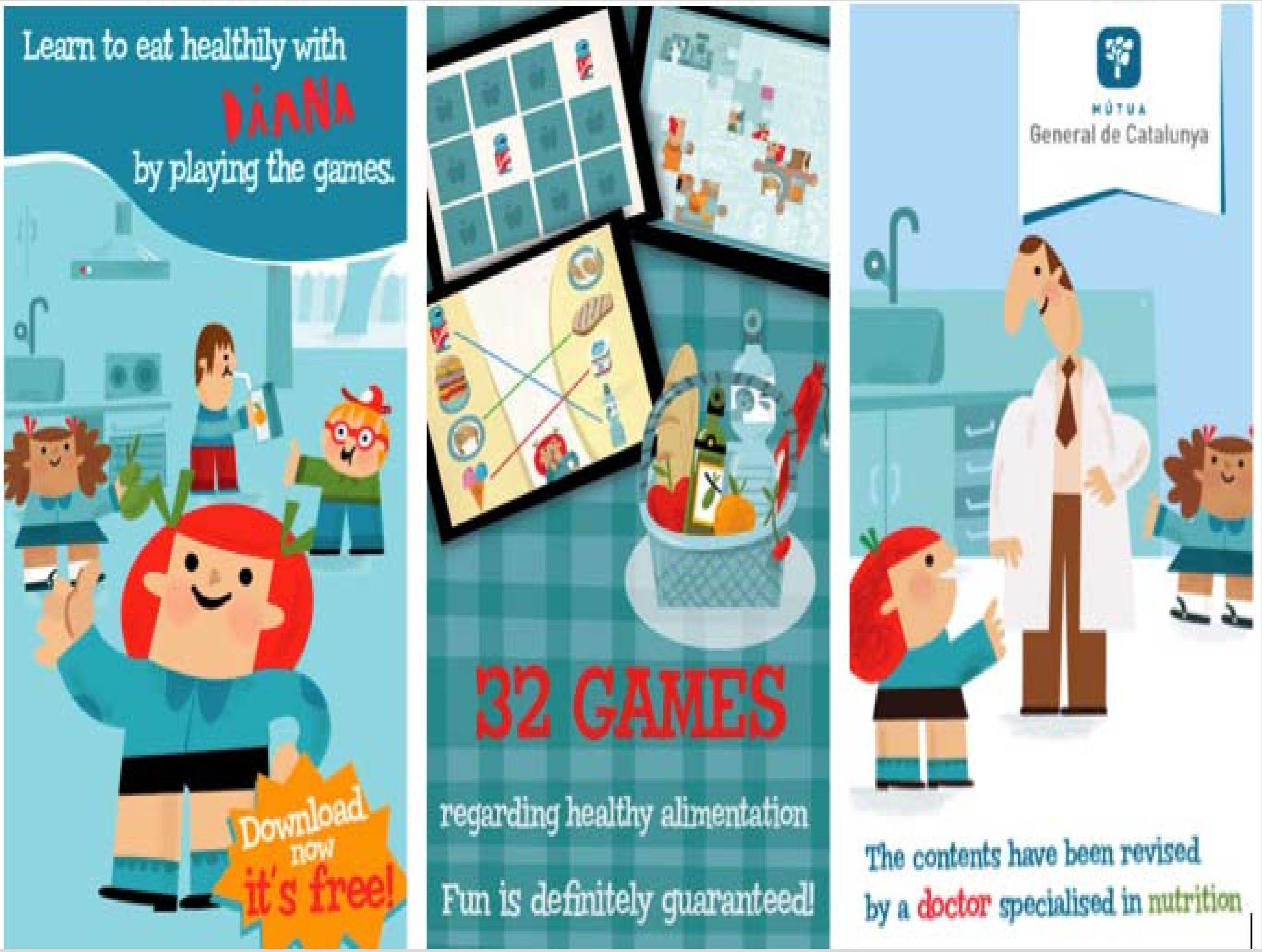

- These apps teach individuals how to do something they don't know. For example, educating children about healthy eating.

10. Badges and rewards

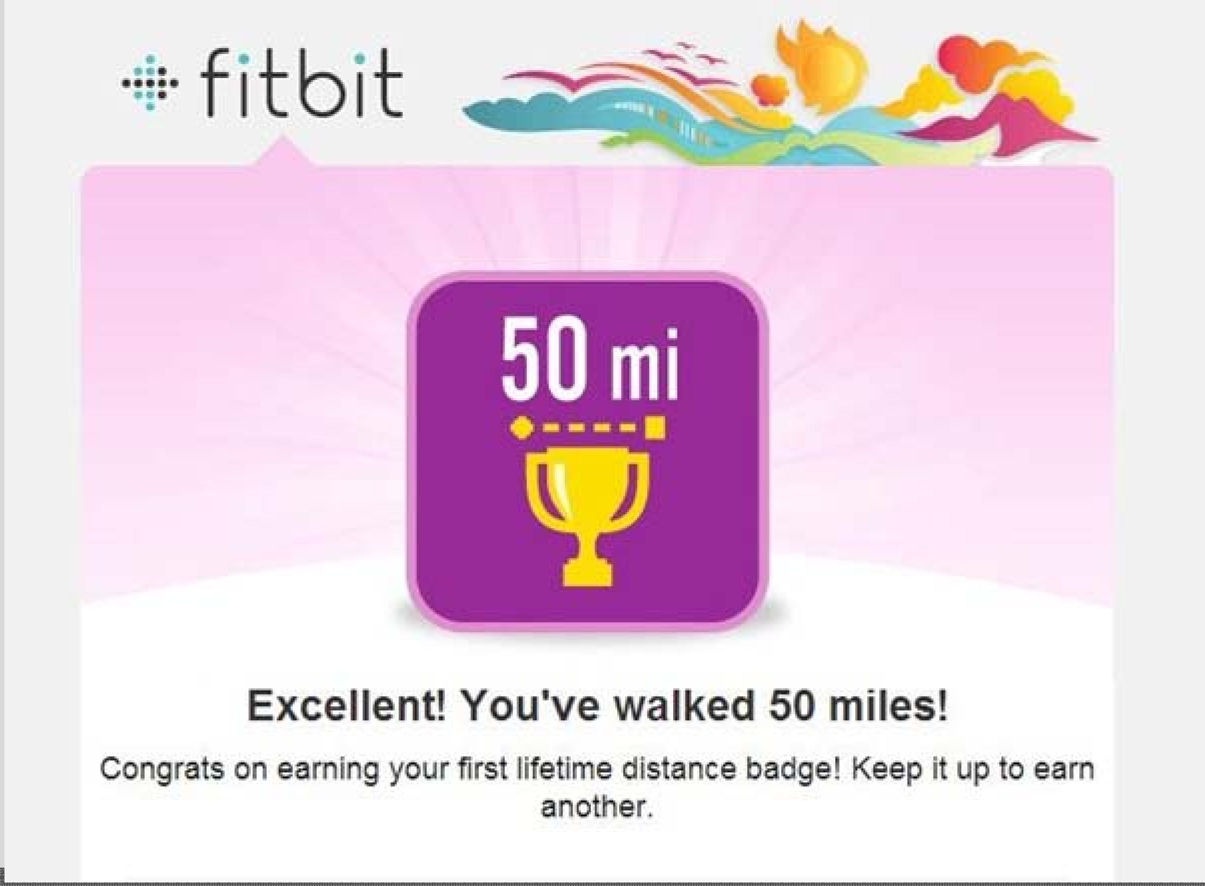

Supplement: Additional file 3: — Trigger materials. (PDF 873 kb) [file 12889_2016_3808_MOESM3_ESM.pdf]
